# Satellite DNA in *Vicia faba* is characterized by remarkable diversity in its sequence composition, association with centromeres, and replication timing

**DOI:** 10.1038/s41598-018-24196-3

**Published:** 2018-04-11

**Authors:** Laura Ávila Robledillo, Andrea Koblížková, Petr Novák, Katharina Böttinger, Iva Vrbová, Pavel Neumann, Ingo Schubert, Jiří Macas

**Affiliations:** 1 Biology Centre of the Czech Academy of Sciences, Institute of Plant Molecular Biology, České Budějovice, 37005 Czech Republic; 2 University of South Bohemia, Faculty of Science, České Budějovice, 37005 Czech Republic; 3 Leibniz Institute of Plant Genetics and Crop Plant Research (IPK), 06466 Gatersleben, Stadt Seeland, Germany

## Abstract

Satellite DNA, a class of repetitive sequences forming long arrays of tandemly repeated units, represents substantial portions of many plant genomes yet remains poorly characterized due to various methodological obstacles. Here we show that the genome of the field bean (*Vicia faba*, 2n = 12), a long-established model for cytogenetic studies in plants, contains a diverse set of satellite repeats, most of which remained concealed until their present investigation. Using next-generation sequencing combined with novel bioinformatics tools, we reconstructed consensus sequences of 23 novel satellite repeats representing 0.008–2.700% of the genome and mapped their distribution on chromosomes. We found that in addition to typical satellites with monomers hundreds of nucleotides long, *V*. *faba* contains a large number of satellite repeats with unusually long monomers (687–2033 bp), which are predominantly localized in pericentromeric regions. Using chromatin immunoprecipitation with CenH3 antibody, we revealed an extraordinary diversity of centromeric satellites, consisting of seven repeats with chromosome-specific distribution. We also found that in spite of their different nucleotide sequences, all centromeric repeats are replicated during mid-S phase, while most other satellites are replicated in the first part of late S phase, followed by a single family of FokI repeats representing the latest replicating chromatin.

## Introduction

Satellite DNA (satDNA) is a class of repetitive DNA characterized by its genomic organization into long arrays of tandemly arranged units called monomers. It is best distinguished from other tandemly repeated sequences by its formation of much larger arrays spanning up to megabases in length and often forming blocks of heterochromatin that appear as nuclear chromocenters and chromosomal bands. Although monomer sizes of 135–195 bp and 315–375 bp, corresponding to the length of DNA wrapped around mono- and di-nucleosome particles, were found to be predominant^1^, the satellite monomers can range from lengths typical for microsatellites (2–7 bp) and minisatellites (tens of bp)^2^ up to over five kilobases^3^. Except for the specific types of tandem repeats including rRNA gene arrays and telomeric motifs that have coding or structural roles^4,5^, the function of satDNA in the genome is still a matter of debate. It has been proposed that satellite repeats may have a structural role in the genome^6^ and that they affect expression of nearby genes by epigenetic modifications induced by specific changes in the environment^7^. Perhaps best documented is the frequent association of satellite repeats with centromeres, implying their importance for centromere determination or function^8^. On the other hand, it has been shown that neocentromeres may arise at satDNA-free regions^9^, and some established centromeres may be free of satellite repeats^3^. Thus, it is yet to be established whether satDNA is a key functional component of centromeric regions or whether centromeres merely provide favorable conditions for satDNA accumulation.

SatDNA belongs to the most dynamic components of eukaryotic genomes, and its high evolutionary rate results in considerable sequence diversification. Therefore, most satellite repeat families are species- or genus-specific^1^. However, precise molecular mechanisms leading to this rapid turnover and their eventual regulation in individual species have not yet been elucidated. A variety of mechanisms have been proposed to generate short arrays of tandem repeats that may provide a template for further expansion. Such mechanisms include unequal crossing over of random sequences^10^, slipped-strand mispairing^11^, and sequence-directed mutagenesis^12^. Tandem duplications of varying length can also result from aberrant replication and replication stress^13–15^. In addition, satellite repeat arrays were found to originate from amplification of short tandemly repeated regions present in rDNA intergenic spacers and 3′ untranslated regions of Ty3/gypsy LTR-retrotransposons^16,17^. Regardless of the primary origin of short tandemly repeated loci, it is supposed that there are additional mechanisms that mediate their expansion into long arrays and subsequent concerted evolution of monomer sequences resulting in their genome-wide homogenization^18–20^. One of the potential mechanisms mediating amplification and sequence homogenization of satellite DNA is recombination-based formation of extrachromosomal circular DNA (eccDNA) from tandem repeats which in turn could serve as a template for their rolling circle replication and subsequent re-integration of the products. Although populations of eccDNA molecules derived from satellite repeats were successfully detected in a number of plant species^21,22^, the evidence for their amplification and re-integration into the genome is still missing. Other potential mechanisms of satDNA amplification include unequal chromatid exchange^10^ and segmental duplication^23^.

To gain better insight into the biology of satellite repeats, comprehensive analysis of sequence diversity, abundance, and homogenization of satDNA families within and between species is needed. In spite of the relatively long history of satDNA investigation, such knowledge is still limited in several ways. Recently introduced methodologies utilizing a combination of next-generation sequencing (NGS) with appropriate bioinformatics tools revealed that previously used experimental approaches suffered from relatively low sensitivity, resulting in efficient identification of only the most amplified satellite repeats with specific properties of their sequences. For example, the very discovery of satDNA was achieved by density gradient centrifugation, whereby it was revealed as satellite bands formed due to the different buoyant density of satellite repeats compared to the bulk of genomic DNA^24^. Alternatively, satellite repeats were often identified based on the presence of conserved restriction sites in their monomer sequences^25^. Consequently, the satellites lacking these features and those with small proportions in the genome were, in principle, hard to identify. On the other hand, novel sequencing technologies provide deep information about sequence composition of complex genomes of eukaryotes via generation of unprecedented amounts of sequence data. These data can then be utilized by bioinformatic pipelines specifically tailored to the identification of satellite repeats from NGS reads without the need for their assembly^26–29^. These approaches have proved to be very efficient and revealed surprising diversity of satellite repeat families in some plant and animal species^29–31^.

In this work, we focus on the characterization of the satellite DNA population in the genome of *Vicia faba*, a species that has long served as a model for cytogenetic studies in plants^32–34^. Owing to its relatively large genome (1 C = 13.41 Gbp) and small chromosome number (2n = 12), *V*. *faba* chromosomes are large and easy to investigate with cytogenetic techniques. Consequently, a number of features like bands corresponding to different types of chromatin and epigenetic modifications have been revealed; however, only a few are associated with specific genomic sequences^35,36^. In our previous study of the repeat composition of species from the legume tribe *Fabeae*^31^, *V*. *faba* was found to carry a large number of satellite repeats that together constituted 935 Mbp (7%) of its genome. Putative satellite repeats were identified based on the properties of cluster graphs obtained by similarity-based clustering of low-pass genome sequencing Illumina reads, as implemented in the RepeatExplorer pipeline^27^. These graphs represent the reads and their sequence similarities as nodes and connecting edges, respectively, and form globular or ring-like shapes in the case of tandem repeats. Such shapes, combined with other properties of the clusters, are reliable indicators of satellite repeats, regardless of their monomer lengths^26,28^. Over 30 putative families of satDNA were identified and partially characterized by these bioinformatics approaches^27,28^, in contrast with only four satellites (FokI, pVf7, a 172 bp-subtelomeric repeat and TIII15) that had been previously reported in this species^37–40^. Here, we provide experimental verification for most of the satellite repeat families predicted by the bioinformatic analysis, their localization on chromosomes, and information about replication timing and association with different types of chromatin. Moreover, we employed ChIP-seq analysis using CenH3 antibody to identify centromeric satellites, revealing their surprising diversity.

## Results

### Large number and sequence diversity of satellite repeats in *V. faba*

Clusters of NGS reads from low-pass sequencing of the *V*. *faba* genome that were classified as putative satellites in our previous study^31^ were inspected manually, as well as by the TAREAN pipeline^28^, to reconstruct consensus monomer sequences from tandem repeats. All novel satellite repeats with an abundance exceeding 0.1% of the *V*. *faba* genome and selected representatives of less abundant satellites (Table 1 and Supplementary Data S10) were subjected to detailed sequence analysis. This analysis focused on AT/GC content, distribution of nucleotides between complementary strands (Table 1), di- and tri-nucleotide frequencies (Supplementary Fig. S1), presence of subrepeats and detection of sequence similarities. In addition, distribution of all selected satellites in the genome was studied by fluorescence *in situ* hybridization (FISH) on mitotic chromosomes. The selected families differed in their nucleotide sequences, and their genomic abundance ranged from 0.008 up to 2.72% of the genome, corresponding to a physical size between 1.1 and 365.1 Mb/1 C. Besides repeat families with a typical monomer length of hundreds of base pairs, there were some with substantially smaller monomers and an unexpectedly large number of 17 families with long monomers ranging from 687 up to 2033 bp (Table 1). The majority of the satellite sequences had an elevated AT content (65–80%), and some were found to have asymmetrical distributions of A/T, C/G, or purine/pyrimidine bases between complementary strands (Table 1).

**Table 1:** Satellite repeats investigated in this study. Novel satellite repeats are numbered with the prefix “VfSat”; references to previously described repeats are given in Notes. Names in square brackets refer to homologous repeats that were partially characterized by Novák *et al*.^28^. The column “ChIP-seq” provides ChIP/input ratios; the values of significant enrichment are highlighted and supplemented with repeat localization determined by FISH. Sequences of all newly identified satellites are provided in Supplementary Data S10.

Satellite	monomer [bp]	Genomic abundance		Sequence characteristics				ChIP-seq (centromere)	Notes
		[%]	[Mbp/1 C]	% AT	max A/T	max C/G	max Pu/Py		
**VfSat1**	191	2.723	365.1	75.9	1.07	1.88	1.22	1.1	[Vf_TA_11]; similarity to TR-9 from *P*. *sativum*
**FokI**	59 / 57	2.322	311.4	59.3	1.44	2.00	1.11	1.0	^37^
**VfSat2**	~26	1.292	173.2	71.4	2.60	5.00	2.00	0.4	
**VfSat3**	702	0.329	44.1	68.1	1.24	1.13	1.21	0.3	[Vf_TA_39]
**TIII15**	58	0.222	29.8	53.4	1.39	2.86	1.90	1.9	^40^
**VfSat4**	38	0.199	26.7	73.7	1.80	2.33	1.92	0.6	similarity to VicTR-B
**VfSat5**	687	0.187	25.1	77.6	1.16	1.19	1.07	0.3	
**pVf7**	169	0.182	24.4	55	1.07	1.00	1.04	0.3	^38^
**VfSat6**	50	0.132	17.6	64	1.91	1.57	1.78	**103**.**6 (CEN1)**	similarity to TR-5 from *P*. *sativum*
**VfSat7**	44	0.102	13.7	70.5	1.21	1.17	1.2	**103**.**2 (CEN1)**	
**VfSat8**	2033	0.061	8.1	71.7	1.68	1.03	1.46	**91**.**3 (CEN4)**	
**VfSat9**	963	0.055	7.4	77.6	1.02	1.77	1.00	0.2	
**VfSat10**	1762	0.042	5.7	74.7	1.08	1.27	1.00	**41**.**2 (CEN1)**	
**VfSat11**	1619	0.040	5.3	75.7	1.08	1.23	1.01	0.3	
**VfSat12**	1004	0.038	5.1	74.2	1.12	1.27	1.02	0.2	
**VfSat13**	47	0.036	4.8	68.1	2.56	1.14	1.76	**149**.**2 (CEN5)**	
**VfSat14**	888	0.035	4.7	75.6	1.06	1.33	1.02	0.3	
**VfSat15**	942	0.035	4.7	76.5	1.11	1.03	1.08	0.3	similarity to TR-20 from *P*. *sativum*
**VfSat16**	1712	0.038	5.1	65.1	1.20	1.00	1.27	**109**.**9 (CEN6)**	[Vf_TA_157]
**VfSat17**	781	0.031	4.2	75	1.11	1.10	1.06	0.4	
**VfSat18**	1172	0.031	4.2	79.7	1.11	1.03	1.08	0.3	
**VfSat19**	1345	0.024	3.3	80.2	1.01	1.35	1.07	0.2	
**VfSat20**	924	0.022	3	74.5	1.23	1.29	1.24	0.7	
**VfSat21**	1057	0.017	2.3	74.3	1.02	1.35	1.10	0.2	
**VfSat22**	1834	0.016	2.2	71.8	1.25	1.22	1.11	0.3	
**VfSat23**	1325	0.008	1.1	73.3	1.17	1.41	1.18	**81**.**9 (CEN2)**	

**Figure 1:**
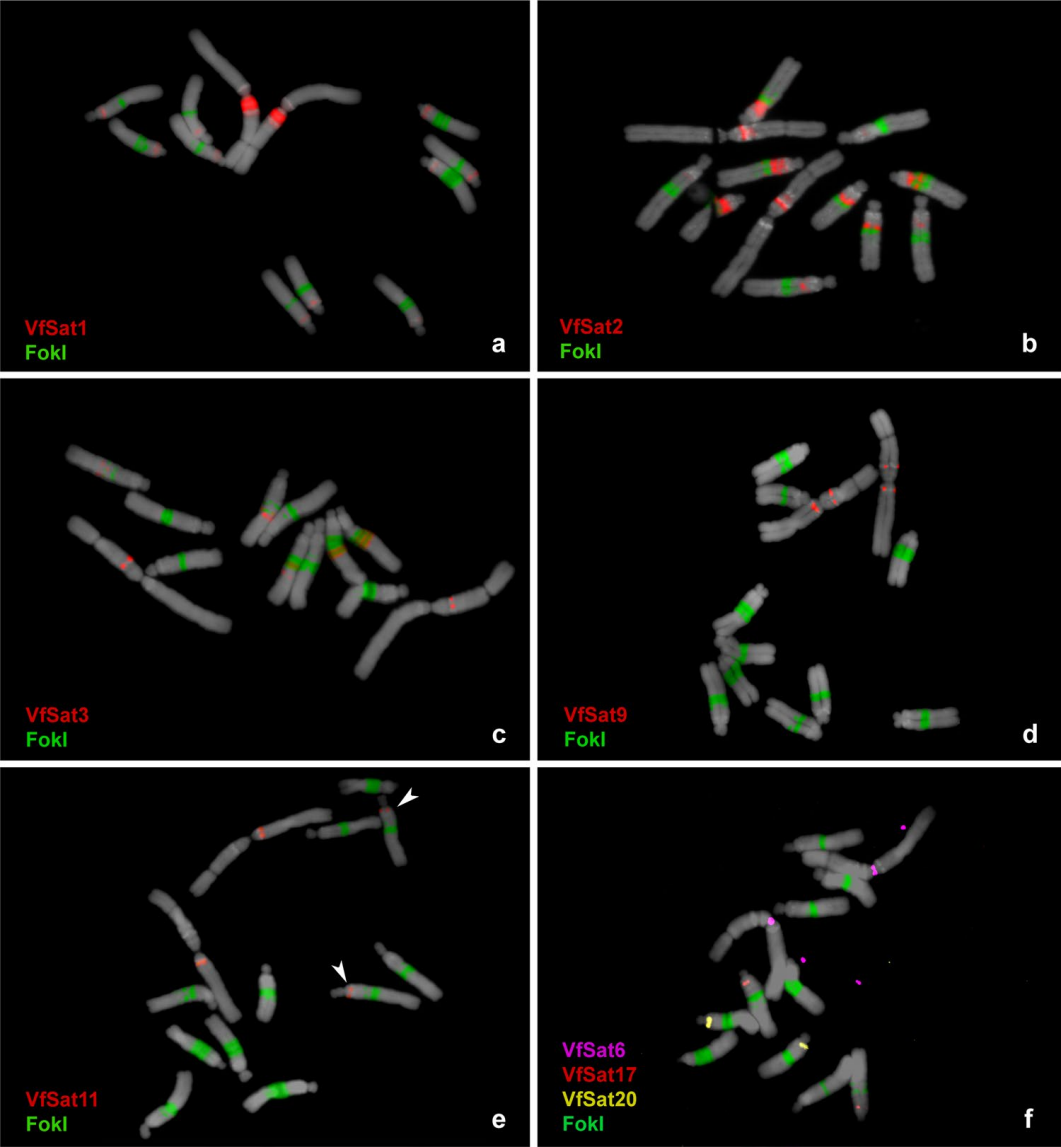
Distribution of selected satellite repeat families on metaphase chromosomes of *Vicia faba*. Satellites were visualized using multi-color FISH, with individual probes labeled as indicated by color-coded descriptions. Hybridization patterns of FokI repeats (green signals) were used for chromosome discrimination as shown in Supplementary Fig. S3. Chromosomes counterstained with DAPI are shown in gray. Arrowheads in (**e**) point to polymorphic VfSat11 signals on chromosome 2 (see Fig. 3b for comparison).

**Figure 2:**
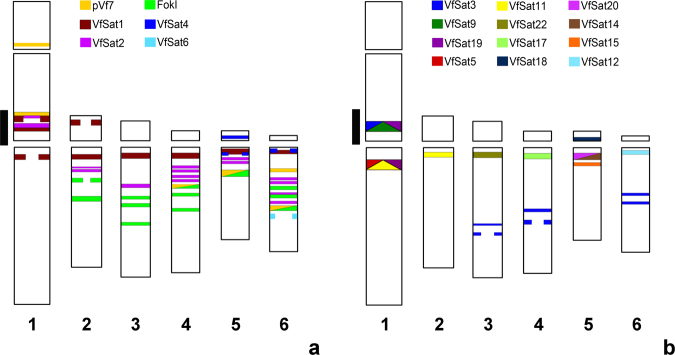
Schematic representation of genomic distributions of all non-centromeric satellites mapped by FISH. Satellites with short monomers are shown in (**a**), while those with long monomers exceeding 600 bp are in panel (**b**). The black line along chromosome 1 marks the region of accumulation of multiple satellite repeats.

There were ten satellite repeats whose abundance exceeded 0.1% of the genome. They included the previously described repeats FokI, TIII15, and pVf7 and seven novel families (Table 1). One of them, VfSat1, was estimated to be of similar abundance to FokI, which is one of the most abundant satellites found in a plant species so far. Contrary to FokI, which is located in a number of bands within the long arms of all five acrocentric chromosomes, FISH with VfSat1 probe produced one major band near the centromere within the satellite arm of the metacentric chromosome 1 and additional minor signals in the pericentromeric region of all acrocentrics (Fig. 1a). VfSat1 was found to share 78% sequence similarity with the pea (*Pisum sativum*) satellite TR-9 (Supplementary Fig. S2), which occurs in terminal regions of three pairs of pea chromosomes^41^. The third most abundant satellite, VfSat2, had a prevailing monomer sequence TATTTGAC(GTT)6, which probably originated from a degenerated simple sequence repeat, (GTT)_n_. Due to its simple sequence, this satellite showed high strand asymmetry values (Table 1). VfSat2 produced FISH signals that were partially co-localized or interlaced with FokI repeats except for an additional band adjacent to a heterochromatic DAPI-positive segment on chromosome 1 (Figs 1b and 2). There was another highly abundant satellite, VfSat3, with similar distribution to FokI, which also occurred in the same additional locus on chromosome 1 as VfSat2, but in this case its signal matched the position of the DAPI-positive band (Fig. 1c). Considering the presence of additional three satellites (pVf7, VfSat9, VfSat19; Fig. 2), this region of chromosome 1 together with heterochromatic loci within long arms of acrocentric chromosomes can be considered a hotspot of satellite DNA accumulation.

### Satellites with large monomers are predominantly located in pericentric regions

A substantial fraction of putative satellite repeats (17 out of 26) had estimated monomer sizes between 687 and 2033 bp, thus being significantly larger than the previously reported preferred monomer length of 135–375 bp^1^. To validate the predicted monomer sequences and confirm their tandem arrangement, PCR was performed with *V*. *faba* genomic DNA as a template and with primers designed to face outwards from the reconstructed monomer consensus. In this arrangement, the amplification can take place only between the primer pairs located in adjacent tandemly repeated monomers (Supplementary Fig. S4). All 17 putative repeats tested using this assay produced the expected amplification products, and their cloned sequences matched the predicted consensus with 92–99% similarity. Dot-plot analysis of the monomer sequences did not reveal any internal subrepeats that could explain their large size as a result of evolution via higher-order repeat intermediates. The only exception was VfSat8 which displayed irregular internal subrepeats (Supplementary Fig. S5). The analysis did not detect any significant similarities between different satellite repeats.

**Figure 3:**
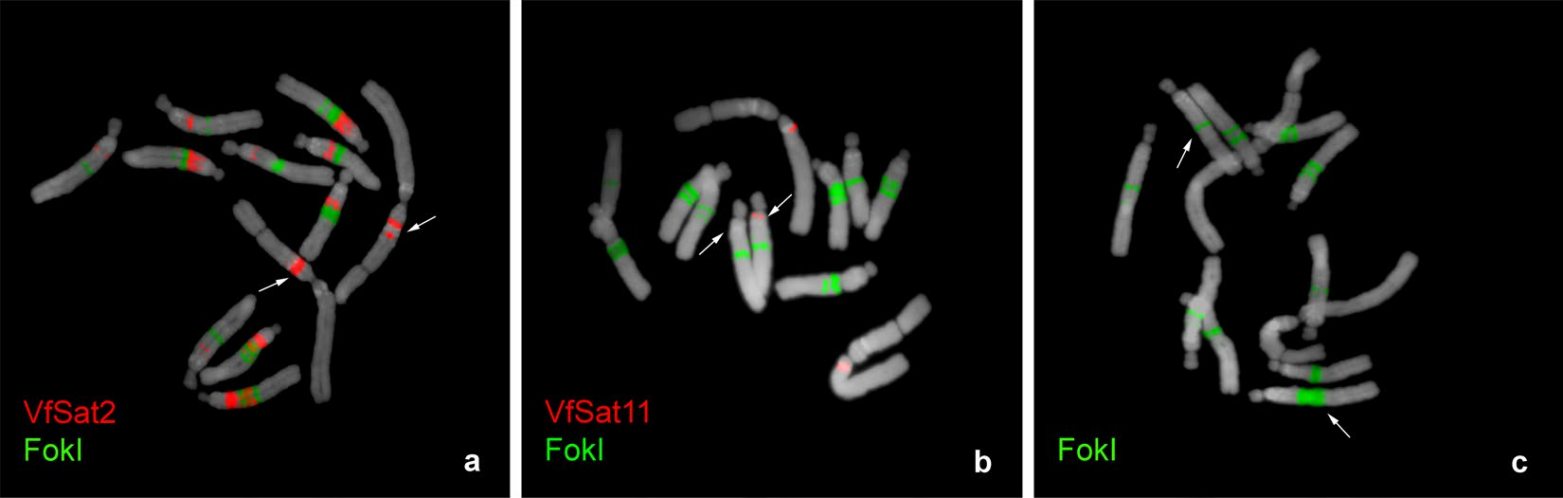
Variation in the number (**a**,**b**) or size (**c**) of FISH signals between homologous chromosomes detected for VfSat2, VfSat11, and FokI. Arrows indicate the positions of polymorphic loci on homologous chromosomes.

Selected clones were labeled and used as probes for FISH. Most of these long monomer satellites produced only a single hybridization signal, except for VfSat9 (one signal on each arm of chromosome 1, Fig. 1d) and VfSat11, which, in addition to labeling one locus on the long arm of chromosome 1, produced a hemizygous signal on the long arm of chromosome 2 (Figs 1e and 3b). Although the FISH signals of long monomer satellites occurred on different chromosomes, they were mostly located in similar positions within their long arms, close to the centromeres (Fig. 2b). Four of the repeats were located within primary constrictions, and further analysis confirmed that they represented centromeric satellites (see below).

### Centromeric repeat composition differs between *V. faba* chromosomes

**Figure 4:**
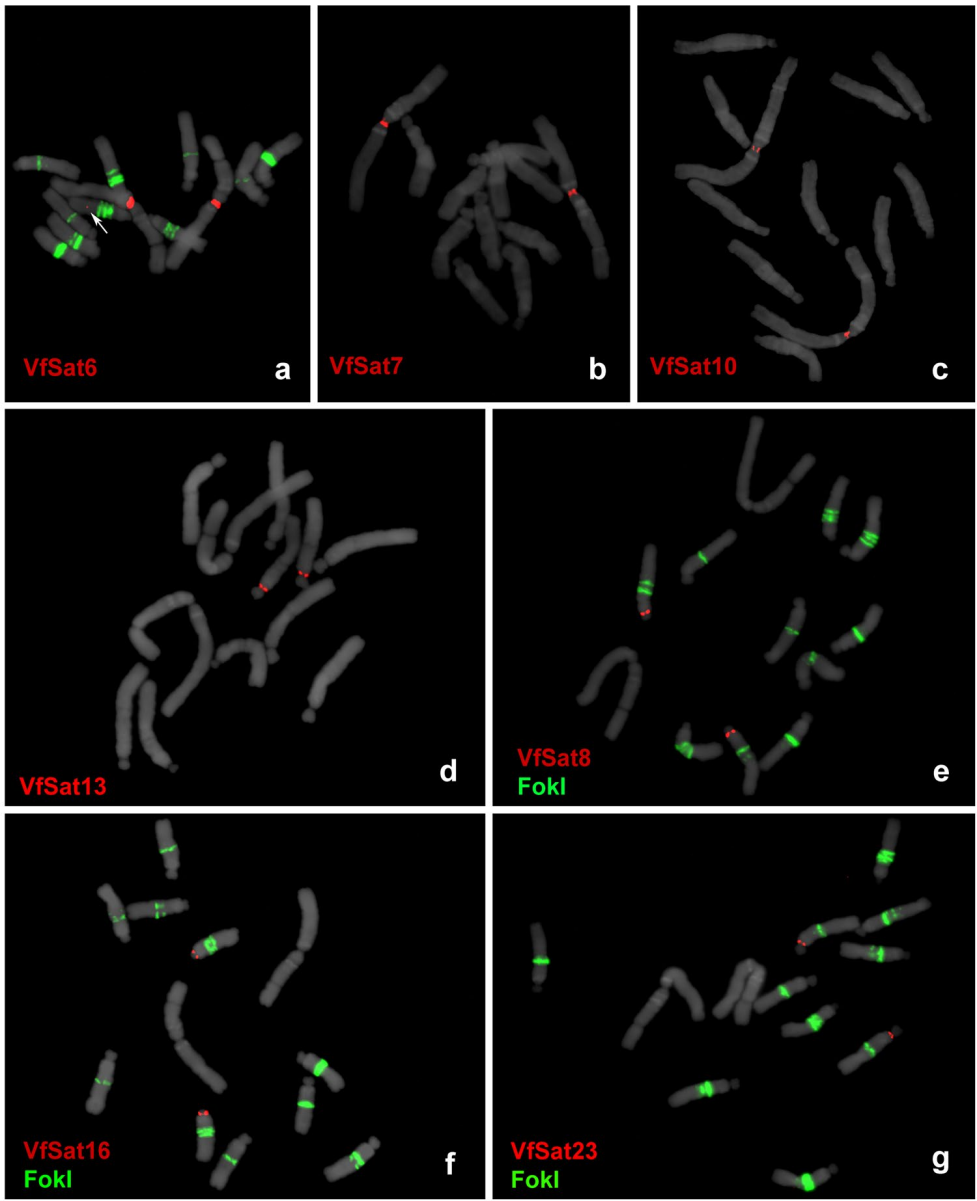
FISH localization of centromeric satellites. Three satellites located in the centromere of chromosome 1 (VfSat6, VfSat7 and VfSat10) are shown separately in panels a–c. Centromeres of four other chromosomes that each contain a single satellite repeat are labeled in panels d–g: (**d**) VfSat13 repeat located in centromere 5, (**e**) VfSat8 in centromere 4, (**f**) VfSat16 in centromere 6, and (**g**) VfSat23 in centromere 2. Arrow in (**a**) points to minor non-centromeric locus of VfSat6 which is detectable only on a fraction of chromosomes. Hybridization patterns of FokI repeats (green signals) were used for chromosome discrimination in (**e–g**). Chromosomes counterstained with DAPI are shown in gray.

The association of repetitive sequences with centromeric chromatin was investigated via chromatin immunoprecipitation using the antibody against the centromeric histone H3 variant CenH3, followed by Illumina sequencing of retrieved DNA (ChIP-seq). The resulting reads were mapped to repeat clusters based on their sequence similarities, as were the reads obtained by sequencing DNA fragments extracted from chromatin preparations prior to ChIP (input control). A total of 21.3 million ChIP and 10.7 million input reads were mapped to reference clusters, and normalized ratios of ChIP to input reads were evaluated for the 500 largest clusters representing highly and moderately repeated sequences with genomic proportions of at least 0.002%. There were seven clusters that showed elevated ratios of ChIP/input reads (41- to 149-fold enrichment), indicative of their association with centromeric chromatin, whereas all other analyzed clusters showed ratios close to or below 1 (Table 1). All ChIP-enriched sequences represented satellite repeats, and their centromeric location was confirmed by FISH (Fig. 4). The seven satellites differed substantially in their monomer length (44–2033 bp), sequence composition, and distribution on chromosomes. Whereas three different satellites were found at the centromere of chromosome 1 (CEN1), four other centromeres contained a single chromosome-specific satellite, and no centromeric repeat was identified for chromosome 3. One of the CEN1 satellites, VfSat6, was found additionally at a non-centromeric locus on the long arm of chromosome 6. However, corresponding FISH signals were very weak and detectable only on a fraction of chromosomes, most likely due to the small size of these loci. No sequence similarities to repeats from other species were detected for *V*. *faba* centromeric satellites except for VfSat6, which was found to be related to the satellite TR-5 (88% similarity, Supplementary Fig. S2) located in the pericentromeric region of chromosome 2 of *Pisum sativum*^41^.

### Three satellites display supernumerary FISH signals or signal size polymorphisms between homologous chromosomes

FISH with the satellite sequences VfSat2, VfSat11, and FokI revealed differences between homologous chromosomes regarding the number of labeled loci or sizes of some signals (Fig. 3). These polymorphisms included VfSat2 loci on chromosome 1, which appeared as either two bands (stronger and weaker) separated by a gap of non-labeled chromatin or as two closely adjacent bands of equal strength (Fig. 3a). The observed genotypes were either homozygous for the former pattern or heterozygous. The other polymorphic site was the locus of VfSat11 on the long arm of chromosome 2, which was missing from one of the homologs in part of the examined individuals (Figs 1e and 3b). In the case of the FokI repeat, an expansion of the signal size was revealed at one locus of the acrocentric chromosome 5, which was paralleled by the size change of a corresponding DAPI-positive band and by an increase in chromosome size (Fig. 3c). The expanded band was observed only in heterozygous configuration, while a part of the genotypes was homozygous for the smaller variant of this FokI band.

### Satellite repeats vary as to their replication time during S phase

**Figure 5:**
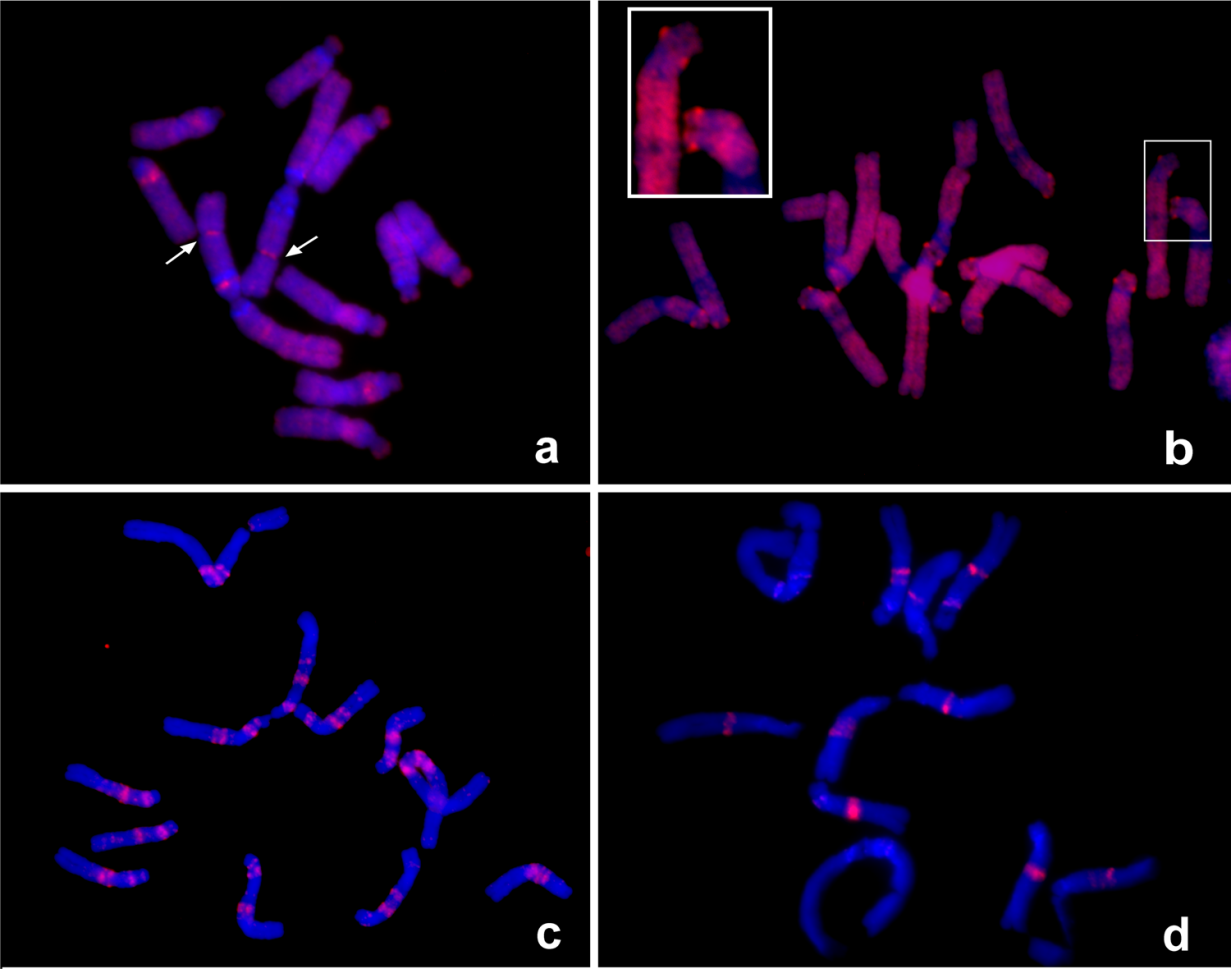
DNA replication assay. Examples of EdU labeling of early (**a**), mid (**b**) and late (**c–d**) replicated chromatin. Arrows in panel (a) show the positions of early replicating NORs. The inset in panel (b) shows a detail of two chromosomes, with bright spots corresponding to labeled centromeres. The two late replication patterns that could be distinguished consisted of labeling most satellite repeats (**c**), followed by exclusive labeling of FokI loci, which represented the last genomic sequences to be replicated (**d**).

The replication time of satDNAs was investigated by incorporating the thymidine analog EdU, employing 15 or 30 min pulses of exposure to EdU followed by fixation of root meristems at various times after the pulse (1–9 h). Depending on the time elapsed since the labeling pulse, the fixed material displayed labeling of early-, middle-, or late-replicating chromatin. Examples of labeled chromosomes are shown in Fig. 5, and the observed labeling patterns are summarized in Supplementary Table S7.

The early replication pattern consisted of weak dispersed labeling with strongly labeled NORs and a few additional bands (Fig. 5a). The early replication pattern was gradually replaced with more uniform staining of whole chromosomes except for their heterochromatic regions. At this stage, corresponding to mid-S phase, there were also distinguishably brighter signals observed at all centromeres (Fig. 5b). The late S phase labeling signals appeared as sharp bands corresponding to most of the satellite repeat loci (Fig. 5c,d). Replication timing of individual satellite repeats was determined by associating their known chromosomal positions with the observed replication patterns and in some cases also by performing their FISH detection on EdU-labeled chromosomes. Taken together, these experiments revealed that the replication timing of *V*. *faba* satellites is not uniform (Supplementary Table S7). Mid-S phase replicating centromeric repeats and VfSat2 preceded most other satellite families, which replicate in late S phase. All these satellites except for FokI finished their replication before the end of S phase, while the FokI sequences alone represented the latest replicating part of the genome (Fig. 5d). The EdU-labeling patterns were correlated with the presence of specific satellite sequences, as exemplified by the FokI bands that were polymorphic between homologous chromosomes 5 and consequently displayed EdU labeling of different intensities corresponding to the sizes of FokI bands. In addition, the earlier replication of VfSat2 was maintained on all genomic loci of this satellite, even those adjacent to the blocks of FokI that were the latest genomic sequences to be replicated (Supplementary Fig. S6). Thus, the replication timing appeared to be sequence dependent, not determined by the chromosomal position. We investigated whether there is a correlation between various characteristics of the nucleotide sequences (monomer length, AT/GC and di- and tri-nucelotide composition) and the replication timing of individual satellites; however, no statistically significant correlation was found.

## Discussion

Satellite DNA still represents one of the most enigmatic components of eukaryotic genomes, which is in part due to the technical difficulties associated with reliable characterization of a representative set of satellite repeats from the genomes of interest. Here we demonstrated that such characterization can be achieved by employing next-generation sequencing combined with bioinformatics tools specifically tailored to this task. Application of this approach to the genome of *V*. *faba* generated a large body of new sequence and cytogenetic information surpassing the evidence that had been gathered so far about satellite DNA in this long used cytogenetic model. The reliability of bioinformatic identification and reconstruction of satellite repeats from NGS data was confirmed experimentally by successful FISH detection of all 25 selected repeats on *V*. *faba* chromosomes. The same strategy was recently used in several plant and animal species, where it revealed surprising diversity of satellite repeats, similar to that reported here for *V*. *faba*^29,30,42^. However, such satDNA diversity is not a common feature of all genomes because there were also species relatively poor in satellite DNA reported after examination using similar approaches^43^, including several species of *Vicia*^31^.

Satellite repeats usually show little or no sequence conservation between different taxa, owing to their rapid evolutionary turnover in the genome^6^. However, great sequence diversity can also be found between the repeats within a single species, as demonstrated here for *V*. *faba*. Except for their preference for AT-rich sequences, the families of *V*. *faba* satDNA did not show any conserved features or sequence similarities, pointing to their independent origin. Despite the wealth of repeat sequencing data from closely related genera of *Vicia*, *Lathyrus*, *Pisum*, and *Lens*^31^, the majority of *V*. *faba* satellites did not show sequence similarities to repeats from other species. This suggests their species-specific origin or rapid sequence diversification. The exceptions included three satellites (VfSat1, VfSat6, and VfSat15) with similarities to satellite repeats from *Pisum sativum*, and VfSat4, which was related to VicTR-B repeats highly amplified in several *Vicia* species^44^ (Table 1 and Supplementary Fig. S2). The existence of rapid turnover of satellite repeats in the *V*. *faba* genome was also supported by the occurrence of polymorphic or supernumerary loci of three satellite repeats, VfSat2, VfSat11, and FokI. This is in agreement with earlier reports of variability of heterochromatic Giemsa bands at chromosomal positions corresponding to FokI repeats observed within and between different accessions and karyotypes^32,35^.

Satellites with long monomers (0.7–2.0 kb) were found to be surprisingly numerous in the *V*. *faba* genome. This contrasts with relatively few cases of such repeats reported to date in other plants. Several satellites with monomers ranging from 0.9 to 4.0 kb were found to accumulate on the B chromosomes of rye (*Secale cereale*)^45–47^. Long monomer satellites were also reported in *Solanum* species, including the Sobo satellite (4.7 kb monomer) of *S*. *bulbocastanum*^48^ and a diverse group of centromeric satellites with monomers up to 5.4 kb from *S*. *tuberosum*^3^ and *S*. *verrucosum*^49^. Most of these satellite sequences display similarities to various retrotransposons or have a complex structure indicating their origin from different genomic repeats^48,49^. These features indicate that the rye and *Solanum* long monomer satellites might be evolutionary young and mostly originate at specific genomic regions represented by dispensable B chromosomes and centromeres. However, none of the repeats reported here for *V*. *faba* had detectable sequence similarity to other genomic sequences, and only a small fraction was located in centromeres. On the other hand, most *V*. *faba* long monomer satellites displayed a preference for pericentric regions, the significance of which is yet to be investigated.

The four satellite repeats with long monomers (1.7–2.0 kb) that were localized in primary constrictions of metaphase chromosomes were proved to be associated with centromeric chromatin using ChIP-seq with the CenH3 antibody. However, the long monomer size was not a universal feature of *V*. *faba* centromeric repeats, as the other three centromeric satellites had extremely short monomers, ranging from 44 to 50 bp. There is mounting evidence that most eukaryotic centromeres are determined epigenetically, independent of the underlying DNA sequence^50,51^. The frequent accumulation of satDNA in centromeric regions is then explained by its positive role in stabilizing centromeres, promoting deposition of inner kinetochore proteins, or simply by passive accumulation due to the absence of recombination-based elimination mechanisms^8,52,53^. Most higher plant species investigated so far have a single or only a few centromeric satellites with monomers hundreds of nucleotides long that are shared by all chromosomes^54^, an observation that is explained by their coevolution with kinetochore proteins^55^. Thus, the monomer length diversity as well as the overall number of different centromeric satellites, all of which are specific to a single *V*. *faba* chromosome, are unusual. Similar diversity has been reported in potato (*Solanum tuberosum*), where part of the centromeres contains chromosome-specific satellites, while the rest is free of satellite DNA^3^. Comparison of homeologous centromeres between potato and its wild relative *S*. *verrucosum* revealed that all but one of the centromeric satellites differ in their sequences, which, together with the absence of centromeric satellites on some chromosomes, indicates that centromeres in these species are evolutionarily novel and still undergo rapid cycles of satellite DNA expansion and contraction/elimination that precede fixation of a single satellite in most centromeres^49^. Even higher intra-specific diversity of centromeric satellites has been described in the pea (*Pisum sativum*), which was found to carry 13 sequence families differing in their genomic abundance and distribution on chromosomes^41^. The genus *Pisum* is closely related to *Vicia*, but chromosomes in *Pisum* and its sister genus *Lathyrus* exhibit a unique morphology of their centromeres, which are composed of multiple separated CenH3 loci arranged along extended primary constrictions^56^. While it was tempting to speculate that the extraordinary diversity of pea satellites originated from the evolutionary shift to its complex centromere structure, the diverse repeat composition of a simple *V*. *faba* centromere calls for the investigation of additional species from both genera to get more representative insight into evolution of their centromeres.

The replication of most *V*. *faba* satellites during the late and the centromeric repeats in mid-S phase is in agreement with observations from other plant species^45,57^. Our data also suggest that, although the replication patterns were found to be conserved for specific satellite sequences regardless of their chromosomal location, the nucleotide sequences alone are not likely determinant of the replication timing, as we did not identify any conserved sequence features among synchronously replicating satellite repeats. Thus, other features like epigenetic modifications of chromatin proteins that were found to correlate with replication timing may be more important^57,58^. Indeed, FokI repeats were previously shown to be distinguishable from the rest of the *V*. *faba* genome by a specific combination of epigenetic marks^36^ which could also explain their partially different replication timing compared to other satellites.

The data described in the present work allow for reexamination of previously described cytogenetic features of *V*. *faba* chromosomes to investigate their eventual correlation with sequence composition and chromosomal distribution of novel satellite repeats. For example, previous studies revealed that the region located proximally on the NOR-bearing arm of chromosome 1 exhibits extreme sensitivity regarding misrepair of DNA damage, in particular after exposure to the mutagens mitomycin C and maleic hydracid, but also to other genotoxins^59,60^. This S phase-dependent misrepair yielded a high frequency of chromatid-type structural aberrations, such as isochromatid breaks, interstitial deletions, duplication deletions, and reciprocal translocations. The clustering of aberration breakpoints was likely due to misrepair of DNA double-strand breaks, which arise from the overlap of excision repair with replication during S phase. The misrepair results from the ligation of the wrong strand ends, favored by sequence homology with the break ends and/or by strand discontinuities at the border between regions with different replication timing^61^. The present study discovered that this aberration hotspot correlates with a region of unusual clustering of diverse satellite repeats, including five repeat families (VfSat1, pVf7, VfSat3, VfSat9, and VfSat19) mapped to two adjacent DAPI- and Giemsa-positive chromatin bands and one (VfSat2) located between them in a DAPI-negative area (Fig. 2). Also, other regions of frequent aberration breakpoints were reported. These were associated with FokI repeats in the middle of the long arms of all acrocentrics, in particular the region on the long arm of chromosome 5 with a largely expanded FokI region^62^. Many FokI loci were found adjacent to or interspersed with VfSat2, the satellite with the most contrasting replication pattern compared to FokI, supporting the idea that such adjacent loci with different replication timing may cause chromosomal instability. Furthermore, due to its chromosomal location, its abundance, and its strong asymmetry of A and T between both strands of the double helix, the non-centromeric VfSat2 may represent the physical basis for the asymmetric bands previously observed by the fluorescence-plus-Giemsa technique after BrdU incorporation for one S phase^34^.

## Methods

### Plant material and DNA isolation

Seeds of the *Vicia faba* cultivar Merkur were purchased from Osiva Boršov (Boršov nad Vltavou, Czech Republic). Total genomic DNA was isolated from young leaves as described by Dellaporta *et al*.^63^.

### Sequence analysis and cloning of satellite repeats

Putative satellite repeats were identified in the course of our previous study^31^ via graph-based clustering of *V*. *faba* genomic shotgun reads using the RepeatExplorer pipeline^27^. Reconstruction of monomer sequences of selected satellites was performed using TAREAN^28^. Reconstructed sequences were used to design oligonucleotide probes for hybridization (Supplementary Table S8) or PCR primers for amplification and cloning of corresponding repeats from genomic DNA (Supplementary Table S9). The latter option was used for satellites with long monomers that could not be efficiently detected using short oligonucleotide probes. The PCR reactions were performed in 30 μL volume containing 1× PCR buffer, 0.2 mM dNTPs, 0.2 μM primers, and 2U of Platinum Taq Polymerase (Invitrogen). The amplification was carried out for 30 cycles of 94 °C for 1 min, 55 °C for 1 min, and 72 °C for 3 min. The amplicons were cloned using the TOPO-TA Cloning Kit for Sequencing (Invitrogen). Plasmid clones were verified by sequencing, and selected inserts were used as probes for *in situ* hybridization experiments. Sequences of all cloned probes were deposited in GenBank under accession numbers MF796528-MF796546.

Over- and underrepresentation of di- and tri-nucleotides in satellite repeats was calculated from unassembled sequence reads according to Burge *et al*.^64^. Correlation between sequence composition and replication timing of satellite repeats was tested using regression analysis in an R programming environment.

### Fluorescence *in situ* hybridization (FISH)

Mitotic chromosomes were prepared from root tip meristems synchronized using 2.5 mM hydroxyurea and 2.5 μM amiprophos-methyl as described previously^56,65^. Probes were labeled with Alexa Fluor 568 or Alexa Fluor 488 (Thermo Fisher Scientific, Waltham, MA, USA) using nick translation^66^. The oligo-probes were labeled with biotin or fluorescein at their 5′ ends during synthesis (Integrated DNA Technologies, Leuven, Belgium). FISH was performed according to Macas *et al*.^67^ with hybridization and washing temperatures adjusted to account for AT/GC content and hybridization stringency allowing for 10–20% mismatches. The slides were counterstained with 4′,6-diamidino-2-phenylindole (DAPI), mounted in Vectashield mounting medium (Vector Laboratories, Burlingame, CA), and examined using a Zeiss AxioImager.Z2 microscope with an Axiocam 506 mono camera. Images were captured and processed using ZEN pro 2012 software (Carl Zeiss GmbH).

### Identification of centromeric repeats using chromatin immunoprecipitation

Chromatin immunoprecipitation was performed with nuclei isolated from fresh leaves as described previously^41^ using a custom-made antibody raised against a peptide designed according to the CenH3 protein sequence identified in *Vicia faba* (CenH3-2_VF)^56^. ChIPed DNA and input DNA control were sequenced on the Illumina platform (Global Biologics, LLC, Columbia, USA) in a single-end, 101 nt read mode. The resulting reads were trimmed to 100 nt by removing the first base and quality filtered to exceed the cutoff quality score of 10 over at least 95 nucleotides. Quality-filtered reads were mapped to reference contigs assembled from clusters of genome shotgun sequencing reads representing *V*. *faba* repetitive sequences produced and characterized in our previous work^31^. Similarity-based mapping of reads to repeat contigs was done using BLASTn^68^ with the parameters “-m 8 -b 1 -e 1e-20 -W 9 -r 2 -q -3 -G 5 -E 2 -F F” and was followed by output parsing to ensure that each read was mapped to a maximum of one repeat cluster with the highest similarity score. The proportion of ChIP and input reads mapped to individual clusters was evaluated to identify repeats with a ChIP/input ratio >10, which were considered to represent repeats enriched in the ChIP sample.

### DNA replication assay

Root tip meristems were treated with thymidine analog 5-Ethynyl-2′-deoxyuridine (EdU) by submersing the roots of three-day-old seedlings for 15 or 30 min in a 10 μM EdU solution in Hoagland medium. The EdU treatment was followed by incubating the seedlings in Hoagland medium for various time intervals ranging from 1 to 9 hours (all incubations were done at 25 °C). Since *V*. *faba* S phase was reported to last 7.5 h, followed by 5 h of G2 phase before entering mitosis^69^, this time sampling enabled observation of metaphase chromosomes with their DNA labeled at various stages of the S phase (late replicating chromatin was labeled in samples collected 1–3 h after EdU treatment while collecting tissues after 6–9 h provided information about early replicating chromatin). Tissue fixation was performed with methanol-acetic acid (3:1) and chromosome preparations were done as described above for FISH experiments. EdU detection was performed using EdU HTS kit (BaseClick GmbH, Neuried, Germany) according to the manufacturer’s protocol, except for the washing procedure, which was done at 35 °C in 2× saline sodium-citrate (SSC) buffer for 5 min, followed by 5 min in 50% formamide/2× SSC, 10 min in 2× SSC, and finally 5 min in 1× BT buffer (0.1 M NaHCO_3,_ 0.05% Tween 20, pH 8.0) at room temperature.

### Accession codes

Cloned sequences of satellite repeats were deposited in GenBank under accession numbers MF796528-MF796546. Raw Illumina reads from ChIP-seq experiment will be available from European Nucleotide Archive under the study PRJEB5241.

## Electronic supplementary material

Supplementary Information S1-S10
