# Supplementary material for: Satellite DNA in Vicia faba is characterized by remarkable diversity in its sequence composition, association with centromeres, and replication timing
Source: Sci Rep. 2018 Apr 11;8:5838. doi: 10.1038/s41598-018-24196-3 (PMC5895790; doi:10.1038/s41598-018-24196-3)
Supplement: Supplementary file 1 — Supplementary Information S1-S10 [file 41598_2018_24196_MOESM1_ESM.pdf]

Satellite DNA in *Vicia faba* is characterized by remarkable diversity in its sequence composition, association with centromeres, and replication timing

Supplementary Information

Laura Ávila Robledillo[1,2], Andrea Koblížková[1], Petr Novák[1], Katharina Böttinger[1,2], Iva Vrbová[1], Pavel Neumann[1], Ingo Schubert[3], and Jiří Macas[1,*]

[1] Biology Centre of the Czech Academy of Sciences, Institute of Plant Molecular Biology, České Budějovice, 37005, Czech Republic

[2] University of South Bohemia, Faculty of Science, České Budějovice, 37005, Czech Republic

[3] Leibniz Institute of Plant Genetics and Crop Plant Research (IPK), 06466 Gatersleben, Stadt Seeland, Germany

[*] corresponding author (macas@umbr.cas.cz)

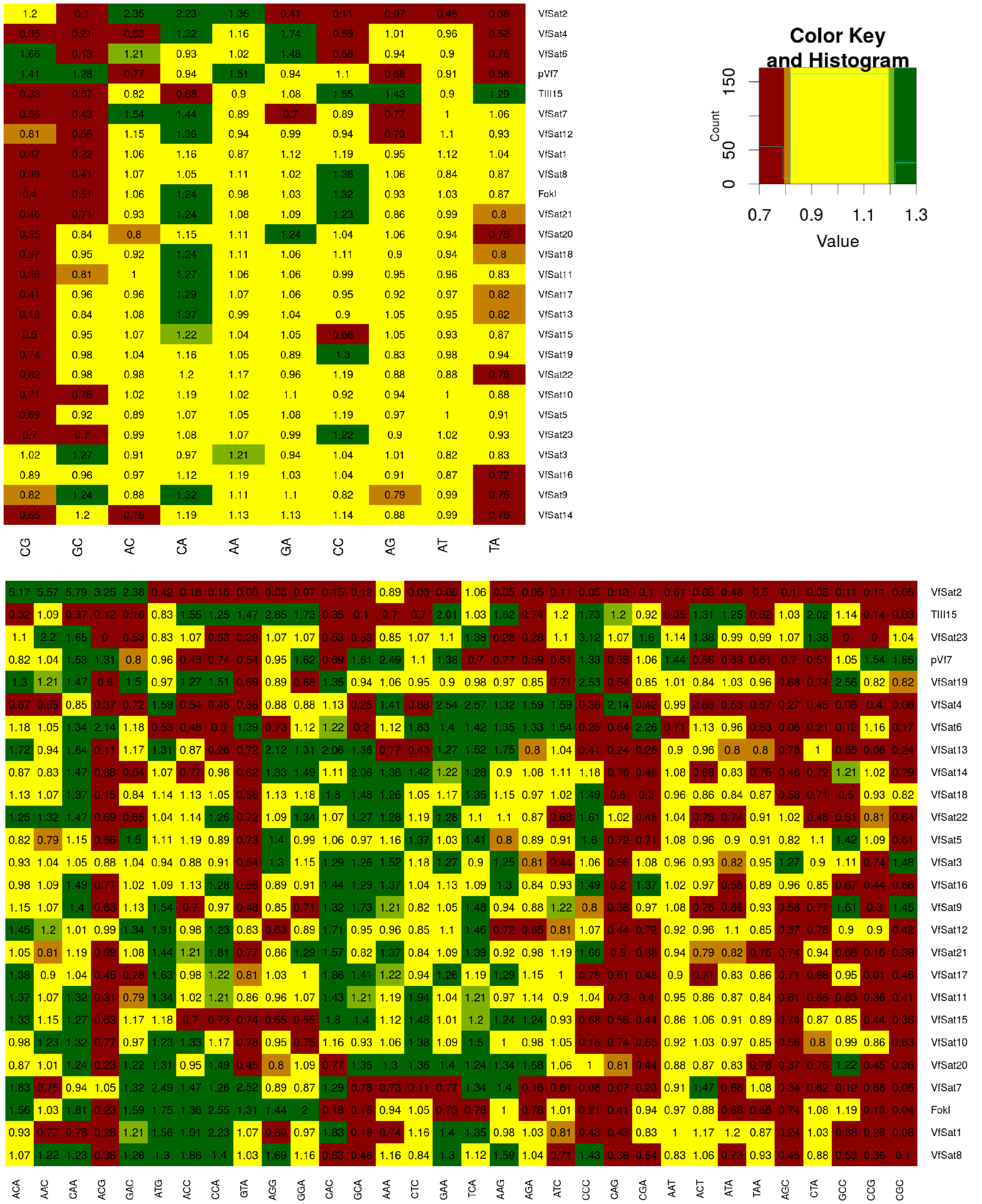

Figure S1. Frequencies of di- and tri-nucleotides in *V. faba* satellite repeats. Representation values ρ^*_{XY} and ρ^*_{XYZ} for di- and tri- nucleotides respectively were calculated from sequence reads according to Karlin and Burge (1995) and are shown by numeric values and colors of the heatmap. A deviation of ρ^*_{XY} and ρ^*_{XYZ} value from 1 reflects marginal (1.20-1.22) or extreme(>1.22) over-representation, or marginal (0.79-0.82) or extreme (<0.79) under-representation.

```
VfSat4          CTGATGAAATTTGAAGTGAACATAAATCTGAAGAAAAT
VicTR-B         CTGATGAAATTTGAAGTGAATATAAGTCTTCAGAAAAT
                ******************** **** ***  *******
```

```
VfSat6          AAGATTTAACACGAACGAGTGTTT-GAATCAATACGGACGAGTAT---CAAAGA
PST_TR5         AATGATTAACACGGACGAGTGTTGAAAATCAATACGGACGAGTATTGACAAAGA
                **   ******** *********   *******************   ******
```

```
VfSat1          CAAATTTTAGGTTACTTCATCACTAAGAAACTAAGTT-AAAAGACTATTACTTAATGACA
PST_TR9         CAAATTTTTGGTTTCTTCATCACTAAGCAACAAAGTTAAAAAAACTATAATAGAATGATT
                ******** **** ************* *** ***** **** ***** *   *****

VfSat1          CATATTCCATATACATTTGAAATAATTCAAATTATCTAATGAGTCTCGATAGTATATTTA
PST_TR9         CATATTATATATAAATGGGTAACAAGTGAAATTACATAATCAATATCAATATTATTTGTA
                ******  ***** **  * ** ** * ******  **** * * ** *** *** * **

VfSat1          TTCACCATATTCATATTGTATTATGGTATAATAGATGTAAACAATTTCAATATTTTTCTT
PST_TR9         TTCGGCATATTCACATTGTATTATGGTATATTATATGTAGATAAATTCAATAAGT---TT
                ***  ******** **************** ** ***** * ** *******  *   **

VfSat1          CTTCTCCATCAC
PST_TR9         CTTCTACATCAC
                ***** ******
```

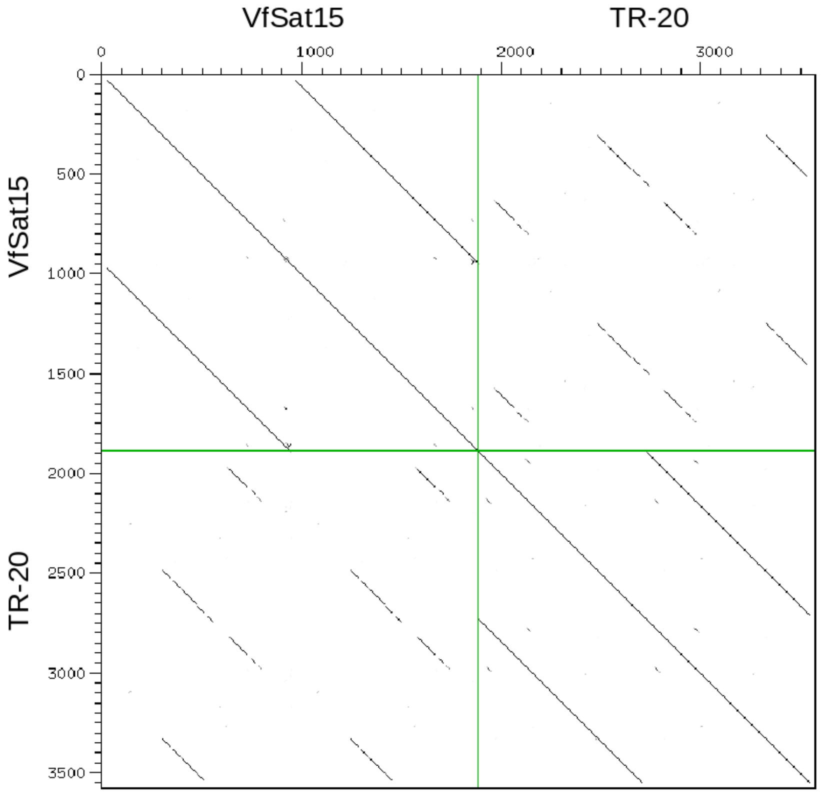

Figure S2. Sequence similarities of *V. faba* satellites to repeats from *Pisum sativum* (PST_TR5, PST_TR9 and TR-20) and *Vicia sativa* (VicTR-B). The similarities are shown as pairwise alignments of consensus monomer sequences, except for VfSat15/TR-20 which are due to long monomers and only partial similarity compared using dot-plot of dimer sequences with similarity threshold of 70 identities over 100 nucleotides.

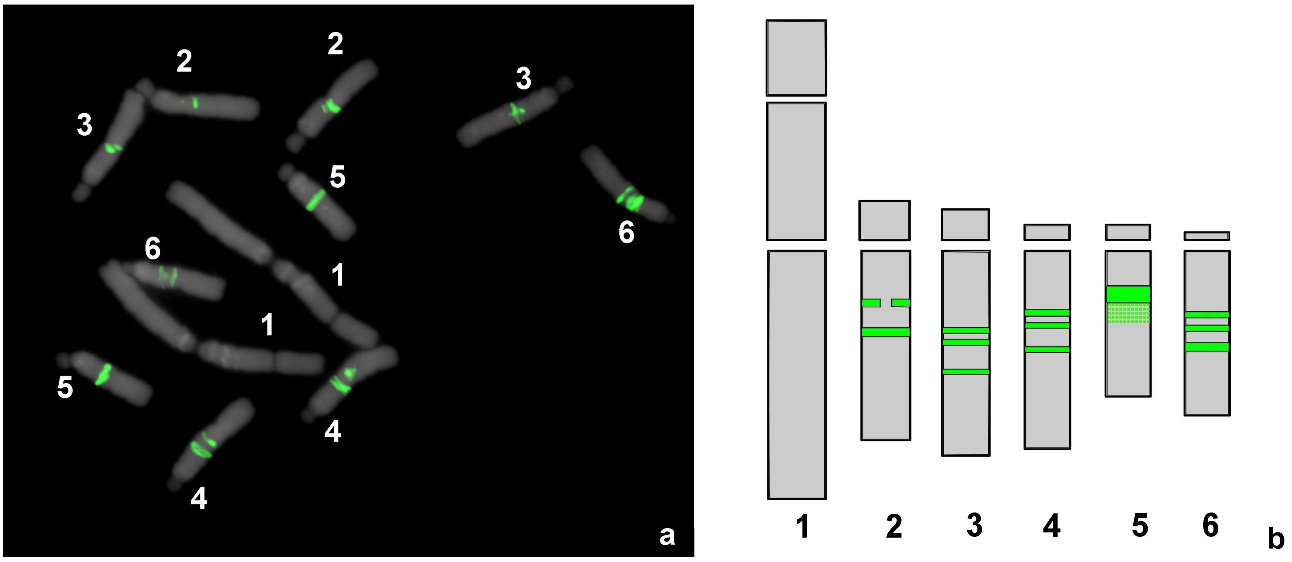

Figure S3. Discrimination of individual *V. faba* chromosomes based on their morphology and distribution patterns of FokI repeat. (a) FISH labeling of FokI repeats (green) on metaphase chromosomes. **(b)** Schematic representation of chromosome morphology and FokI patterns. The polymorphic FokI band on chromosome 5 is marked with dotted pattern.

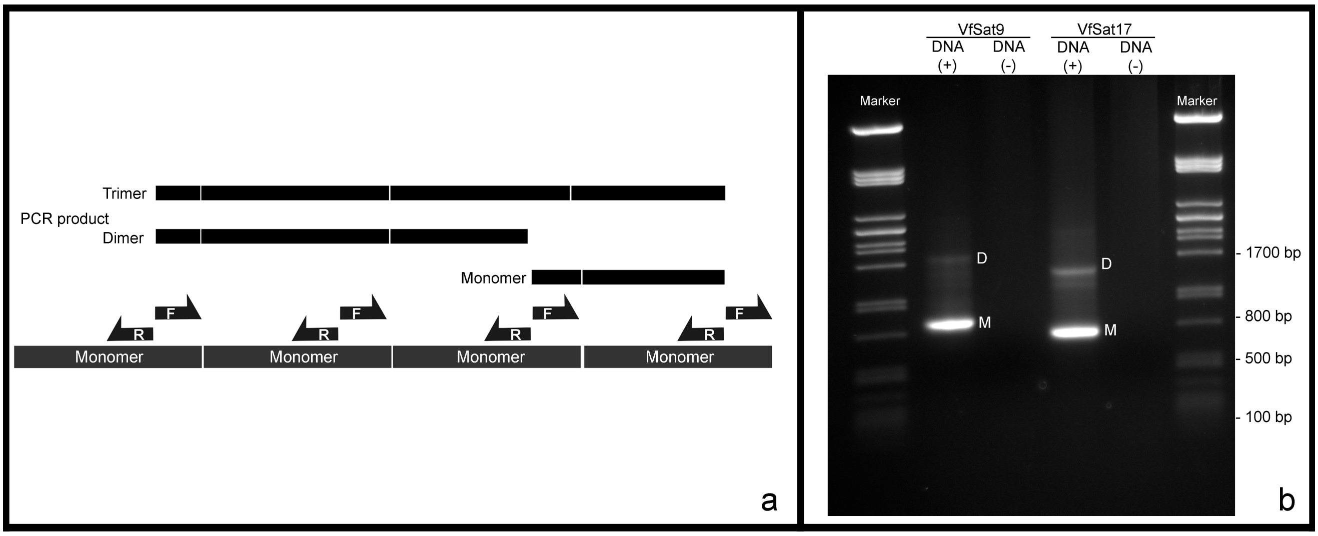

Figure S4. Design of primers used for PCR amplification of satellite repeats from genomic DNA. (**a**) Forward (F) and reverse (R) primers were facing outwards from predicted repeat monomers, thus generating amplification products only when their target sequences were arranged in tandem. (**b**) Example of agarose gel electrophoresis of amplification products from *V. faba* genomic DNA using primers for VfSat9 and VfSat17 repeats, showing bands corresponding to amplified monomer (M) and dimer (D) sequences.

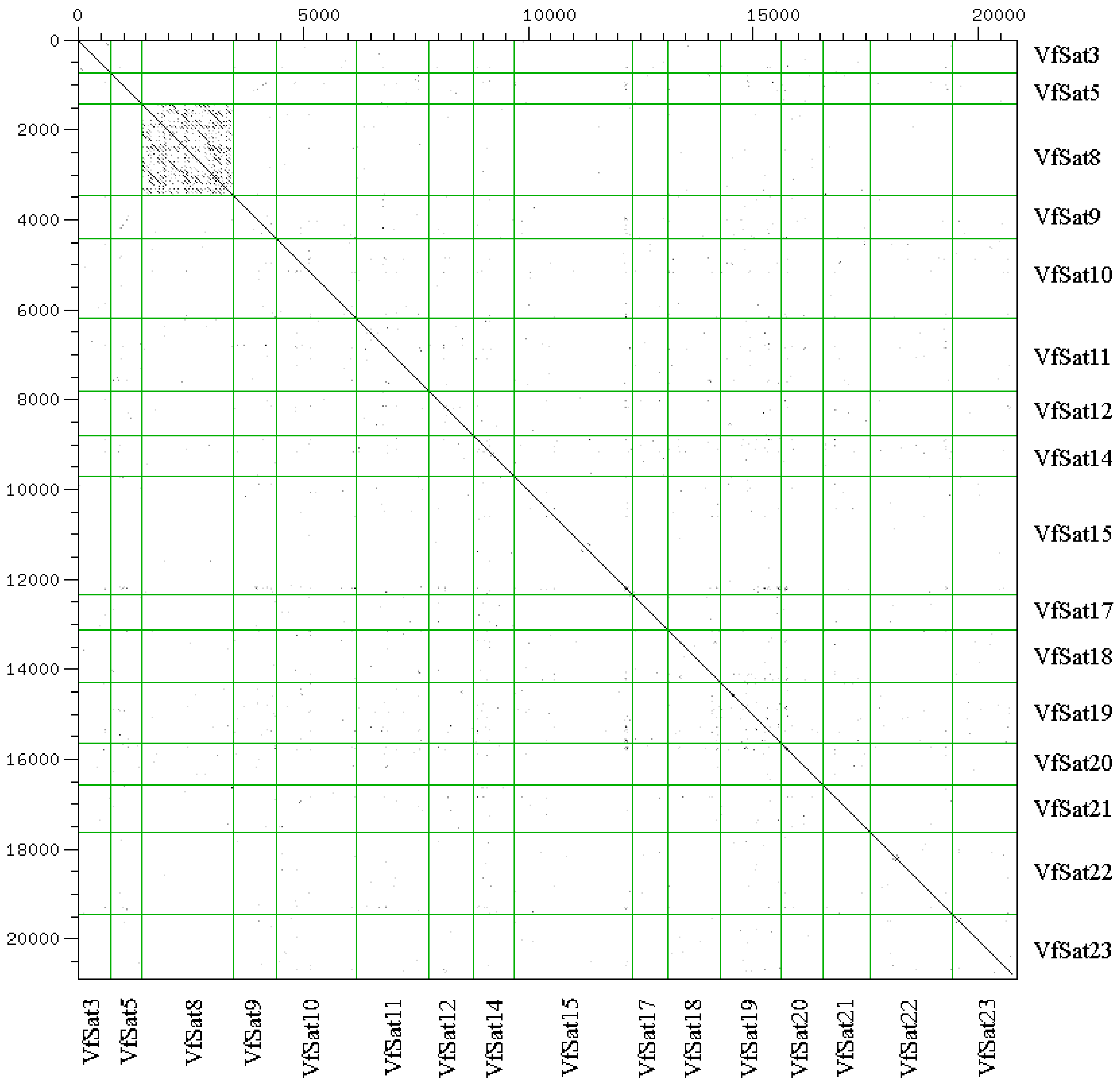

Figure S5. **Dot-plot comparison of satellites with long monomers.** Similarity threshold of 70 identities over 100 nucleotides was employed.

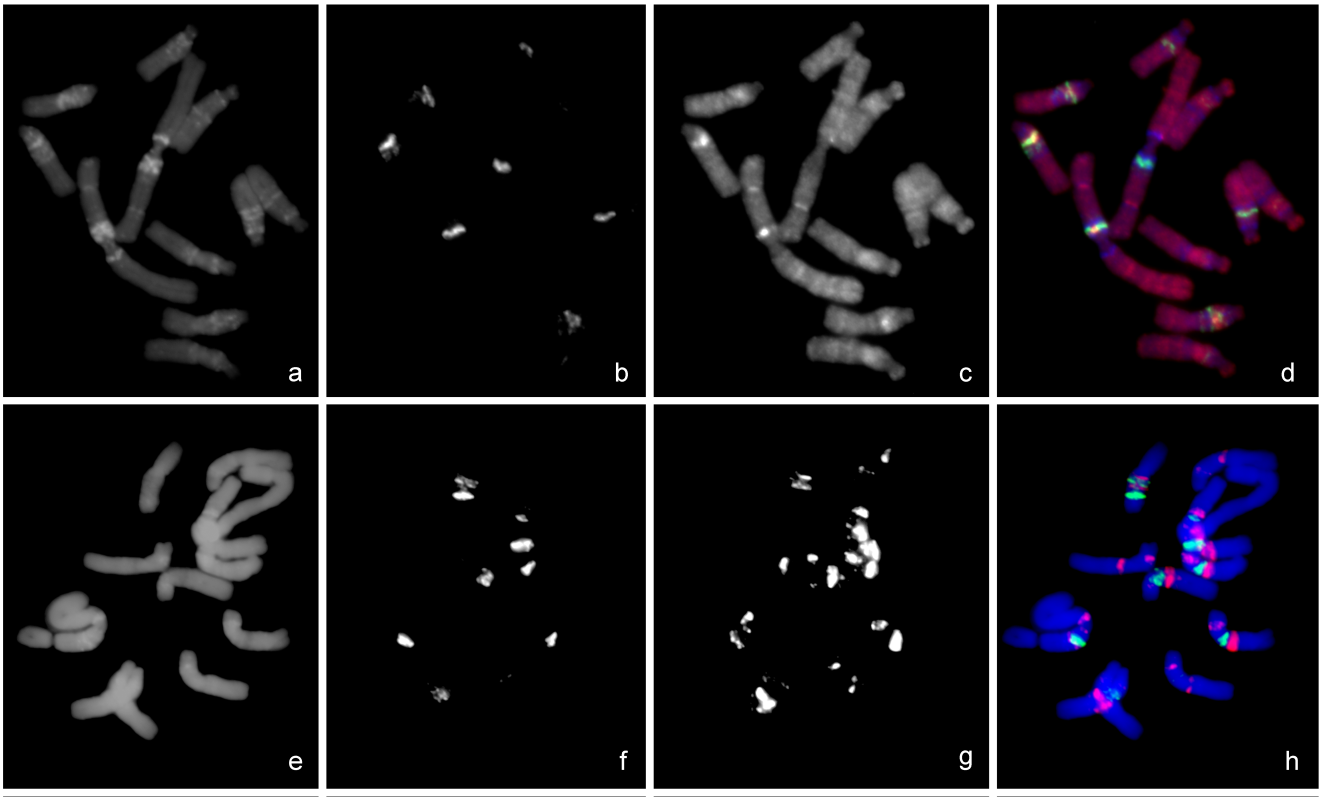

Figure S6. Combination of replication assay (EdU labeling, red) showing early (**a-d**) and late (**e-h**) replication patterns with FISH detection of VfSat2 (green). Chromosomes counterstained with DAPI are shown in blue.

Table S7. Replication timing of satellite repeats.

	Replication time (hours since the start of S phase)								
	**1**	**2**	**3**	**4**	**5**	**6**	**7**	**8**	**9**
VfSat2 included in dispersed labeling pattern*	+	+	+	+	(+)	(+)	-	-	-
Centromeric satellites	-	-	-	(+)	+	+	-	-	-
VfSat1	-	-	-	-	(+)	+	+	-	-
All remaining satellites	-	-	-	-	-	(+)	+	(+)	-
FokI	-	-	-	-	-	(+)	+	(+)	(+)

* VfSat2 did not replicate during late S phase (hours 7-9) but its exact replication timing was not determined due to the lack of distinct patterns on the background of dispersed labeling during early/mid S phase

Table S8. Sequences of oligonucleotide FISH probes.

ID	Probe	Label	Sequence 5'->3'
**VfSat1**	VFBm2H1	Biotin	CTTTTAACTTAGTTTCTTAGTGATGAAGTAACCTAAAATT TGGTGATGGA
**FokI**	VFBm3_Fok_H1	Fluorescein	CTACCTTCCATAATGACAAGGCTACCATCCATTGGAGTAA CAAAAATCTC
**VfSat2**	VFBm15H1	Biotin	CAACAACAACAACAACAACGTCAAATAAACAACAACAA CAACAACAAC
**VfSat5**	VFBm105H1	Biotin	AGCTCCCATCATCCAAGTAGGTAGTGCTATCTCACTCCT
**pVf7**	pVF7_TA_CL34	Biotin	TAAACCGAGGGCTTGTCGAAACGCTACGAAACTTTGGG GACACTCTCAAT
**VfSat6**	VFBm127H1	Biotin	ATCAAAGAAAGGTTTAACACGRACGAGTGTTTGAATCA ATACGGACGAGT

Table S9. PCR primers used for amplification of satellite repeats from genomic DNA and GenBank accession numbers of cloned probes.

Satellite	Primer	Sequence 5'->3'	Accession number
**VfSat3**	Vf_TA_CL39_1<br>Vf_TA_CL39_2	AGCACGAATAAAACTAAAGTTC<br>TACTTTTGAAGTGAAATGGAG	MF796528
**VfSat4**	VFBm102c102F<br>VFBm102c102R	GCAGAAAATCTGATGAAAAATGATG<br>TTGTTCACTTCAAATTTCGTCAG	MF796529
**VfSat7**	VFBm144c57F<br>VFBm144c57R	TACCATAATGAATGGACCTTTATACT<br>CGTTACATATTTTGACTAAGTACTTTTAATATG	MF796530
**VfSat8**	VFBm164c16F<br>VFBm164c16R	CTAATCATGTTATGTCTCATGTAGTTTC<br>GAAATGTTAATATCTTGTTAATCAAAGACT	MF796531
**VfSat9**	VFBm168c23F<br>VFBm168c23R	CTATTTTCAAATGTATATTCGACATGC<br>TAGGCCTTTTAGAATCAGTTATTGACA	MF796532
**VfSat10**	VFBm186c4F<br>VFBm186c4R	AGGAAACAAATAACATTGCATTCTC<br>ATTTTTACCGTCTCTACAAAGATTGAT	MF796533
**VfSat11**	VFBm187c11F<br>VFBm187c11R	CCAAAACAATAACAAACAACATCAA<br>CTTATGTTGTTTAGCGACATTGGA	MF796534
**VfSat12**	VFBm190c10F<br>VFBm190c10R	TGTGTTTCAGTTCAAATGTGTGTCT<br>AAATGTGAGATAACAACTACGGACA	MF796535
**VfSat13**	VFBm197C23F<br>VFBm197C23R	GGTTATAAAACAACAAGCAAAGTAAG<br>CCTTGCATGTTTCCCTTTAT	MF796536
**VfSat14**	VFBm198c11F<br>VFBm198c11R	CTCTCTGTTCAATTTCTCAATCGTC<br>GATTATATCTGCGAATGCCTGAA	MF796537
**VfSat15**	VFBm199F<br>VFBm199R	TGAGAAGTCGTCCATCCTGA<br>TTGCACAAAGAGAAACTTAAGGAA	MF796538
**VfSat16**	VFBm200C23F<br>VFBm200C23R	ATCAAATTAGTTGGGGCTTG<br>TTCGGCAATCGTAATCAAC	MF796539
**VfSat17**	VFBm205c11F<br>VFBm205c11R	GGTATGAGAATGGTGTATCTTTTATCA<br>AGAAAAGATATTTGGTTTCGAATGA	MF796540
**VfSat18**	VFBm207c9F<br>VFBm207c9R	AAGATTCATCGGAAGTATTCCTTTT<br>GAGAAATCACTTTGTAAAGAATTTGGA	MF796541
**VfSat19**	VFBm220c12F<br>VFBm220c12R	TTCTGCACAAGTAAATGAATGGTTAT<br>GGTTGAAGCCACTTATAAATCTCAA	MF796542
**VfSat20**	VFBm224c8F<br>VFBm224c8R	ACTGGGCAGAAAAATGAGACTTA<br>TTCAACTTTGCAAAAGGGGTTA	MF796543
**VfSat21**	VFBm233c2F<br>VFBm233c2R	CACACTATTGTAATCTCCTTGCAAAT<br>ACAAAATGGGGTAGCATGGA	MF796544
**VfSat22**	VFBm237c7F<br>VFBm237c8R	TCAAATAGGACAACGTATTTAAGCAA<br>TAATGCAGTGTTGTCAATGTTGG	MF796545
**VfSat23**	Vf_TA_CL281_2<br>Vf_TA_CL281_1	TAACCCAAGAGGACCCAATG<br>GATACCTTCCTCACCCATACA	MF796546

Supplementary Data S10 - Reconstructed monomer sequences

```
>VfSat1_TA
CATCACCAAATTTTAGGTTACTTCATCACTAAGAAACTAAGTTAAAAGACTATTACTTAATGACACATATTCCATATACATTTGAAATAAT
TCAAATTATCTAATGAGTCTCGATAGTATATTTATTCACCATATTCATATTGTATTATGGTATAATAGATGTAAACAATTTCAATATTTTT
CTTCTTCTC

>FokI_TA
TCCATCGGAGTAACAAATCTCAACAACGAACTATCTCCCATAATGACGAGACTACCA

>VfSat2_TA
TTTATTTGACGTTGTTGTTGT

>VfSat3_TA
TAATAACTAAAAAGGAGGCAAACTAAATTGGTGGGTGTAATGAAATTTTCGCACAAACAGTAGCGTAGGTCCAAATAAACGCTTTGTCAAC
ACCAAGTATTTTTCTCGAAATCAAACCATTTTCAAAATATTTTCATAACAAATGCACTCGAAGGACACATGCATTAAGTAGTGAAGAAGGA
ATGATTAAATGTATGTCCCTTTGTTCTTAGTCCGACGACGCTGGCTGTATAGGTTATCGTTCGAATGCTCACCTTCACTCATGAACTTTAG
TTTTATTCGTGCTATATACTTTTGAAGTGAAATGGAGGAAAATGACTAGTTAGGCGTATGCTCCTCTTATAAAGATTCTAATAAAGACAAA
GAAGAGTGATCTCTTCTGTCCAAAAGGGTAAACCAACTTTGAAAGAGAAAATAGAGCAACAGACAATATGCCATAAAAGTTATTTAATAGC
ACTTTCTTTTGTTTTTGATAGCGCTGAAAAGCGCTATTAACCGTGCCACTAATGTGAACGATTTTGTTTAATAGCACTTTAAAAGAGCTAT
TAAAGTACTAGTTTTTAATATAAAAATAATAATTAATTAAAACAATAACTAATAAGGAGGCAAACGAAATTTGTTAGGGAAAATGAATATT
TTTTCATAAACAATAGCGTCGGCCTGAACCGTCGCTAATATAAAAAAATAAATAATTATTTAAAA

>TIII15_TA
TACTTCGAAATGAAAGCCTGGATAGTAGGGCTGTGAGAAGGAACCTGGATAGTAGGGT

>VfSat4_TA
CTGATGAAATTTGAAGTGAACATAAATCTGAAGAAAAT

>VfSat5_TA
GTCAATGATATGAGTAAATGGCTATTTCTATAGTTAATGTAATATTTAGTTTATAAATAAATAATTATATCATATTTTCGTTTACGGCCTA
ATTTTGTTCATTAAAACATAAAGGTACTTAGTTAGAATTGAGTTTGATTAAAAATGAATGAGTAAAAGTATGTATGTTTAAAGTATTTAAG
TTTGATTTTTATTTTGTGAGATTTATATTTTTAGTTTAAATAAATTATAAAAATTGCTTTTGAGTAATAAATACGATTAAATTTAGATAGT
AATTTAAATTCTAAAATTTAAAATACTATCATTCATAGTCAAAACTTGATTGAGGGTGAAAAATAAACTATTCAGCATATTTATTCTCATT
GATTTTTCAATTTGTCAATTTATTTAACATTACTTGAATTGTTAATTTACAAATGTTTAAGTGAAATAGACTTTTATATAGGTTTGTAGGC
TAATCAGACATTAAAAATGACTAGGCTCAGATCTACAAATAAGCTTATAATAGATTACCGGTTCATACTTTACATCCTTTATAGATTTAGA
ATTTTTTAGCTCCCATCATCCAAGTAGGTAGTGCCTATCTCACTCCTTCGAATTTTTTATTCCTTGTATATGTGAAAGTCATTAGTATAAT
TTATCATTCATTGAAAAATAAGTCACCTCTCTTTATAATTTGAATCTTCATATGACAG

>pVf7_TA
GAAATTCAAAATAAACCGAGGGCTTGTCGAAACGCTACGAAACTTTGGGGACACTCTCAATGTGTTATTTGAGATGTCCATGCAAAAAATC
AGCAGGAGATTATTTTCCTAAGGCGCGTTTGCCTCCGCTTCCGTTTTTGGCAAAAACGCAATTGCACGCGTACCGTCG

>VfSat6_TA
AAGATTTAACACGAACGAGTGTTTGAATCAATACGGACGAGTATCAAAGA

>VfSat7_TA
ATGTACATTGATGAATGAACCTTTACACTAAGTCAAAGTATGTA

>VfSat8_TA
CGATAAGGTTTAATCCTTTCAAATACTCATAGTATGTATAATATTCCTTTCTTTTCATTAGGTTTATTATGGTTTGAAATTAAGCTTAAAT
GGGACAGGCCTTTCAAATGCTTATACAATGTAACATAGTTTCTTTCTCTTTAATTGGTTTTTAATGACCGAGTATTTGGTCAACATGGATC
GGACCTTTCAGCAAATCATATTATGTAATCTACTTCTTACATGTTTATTTATTTTCTTTCGATTTAGTCTTTGATCAAGAAGCGTTTCTAT
TTTCAACTACTAATTTTATAAGATGCATTTATTTAGTCATCTTTTTTAGTCTAATTACTAGGTTTTTATCCATCAAGGAATGAATATTTCA
ACTACTCAAATCATGTAATGTACTTTATTTCCTAACTATTTGGTTTCCCATGATTATGGCTTTGACCAACCCAGACTAGACATTTCAACTA
CTCATTGTACGTAATGTACTTATTTATTTTTATTTGGTTTATTAGGGTTTAGTCTTTGATTAACAAGATATTAACATTTCAACTAATCATG
TTATGTCTCATGTA
GTTTCTTACTTTTAGTTGGGTTTTAAGGAATAGGTGTTTGGTCAGTAAAGAAGGTACCTTTGAAATACTCTTAGTATGTAAATCACTTATT
TTCTCTTCAATTGCTTCTTTATGGTTGATTCTTGGGTTGATAAGGATTGATTCTTTCAATTACTCATAGTATGTGTAATATTTCCTTTATT
TCCATTTGGTTTATAATGGTTTGAGATTTGTTTTACATGGAAAGGACCTTTAAACTACTCTTATTTTATAGTGTAGTTTCTATCTATTTAT
TTTGATTTTAATGACTAAGACTCTGACCTTTCAACTCATTATATTAGTTTACTACTTCTTACCTATTTATTTGGTTTCTTACCTTTAAGTC
TCTGACCAACAAGGGGTTCACTATTCAATTACTAATGTTATGTAATGTATTTCTTTTATCTTTATTTAGTTTCTAATTACTAGGTCTTTGT
TCATTAAGTAATTGACCTTTCAACTTCTCAAAGTTTGTAATGTACATTCTTTCCTTTTTATTTGGACTCTTAATGTAAGTCTTTGATCTAC
AAGGGTCATACATT
TCAACTTCTTATTATGTGTAATGTACTATGCTTCTCTTTAATTGGTTTTTTAGGGTTATATATATTTTATTAACAAGGTATGAACTTTTCA
ACTACACACATAATGTAATGTAGTTTGTTTCTTTCATTTGGTTTCGGAAAACTATGCCTTTGGTCAATAAAGAAGGAACCTTTTAATTACT
CATAGTATGTAAAGCACGTATTTTTCTTCATTTTCTTCTTTATGGTCGAGTCTTTGTTCGATAAGGTTTGTTCTATTCAACTGATCATAAT
ATGTGTAATATTTCCTTTCTTTTGATTTGGTTTATTATGGTTTGTGTTTGGTTTAATCGGGATGGACATTTCAACTACCAATATTATTTAA
TTAGTTCCTTTCTCTTTATTTGGTTTTTAATAACTAAGTCTTTAGTCAACAAGGACCACATGTTTCAACAATCATATTATGTAAAGTAATT
CTTACATGTTTATTTGGTTTCTTATGATTAAGTCGTTGATCAACAAGGGATTCTCTTTTCAACTACAAATGTTATGTAATGTATTTATTTC
ATCTTCATTTAGTT
```

TCCAAATACTAGTTCTTTGTTCATCAAGGAATGCACCTTTCAACTACACAAATAATGTAATGTACTTTATTTCCTCTTTATTTGGTTTCTT
ATGGTTAAGTCTTTGATAAACAAGAGCCTGGAATTTGGACTAATCATTTTATGTAATGAACTTTTTTTCATCTTTATTTGGTATATTAGGG
TTCACAAGGAAGGACCTTTCAACTACTAATGTTATGTAATGTATTTTATTTCTTTCATTTGATTCCTAATGGCCAGGTCTTTAGTCAACAA
GGAAGGGATATTTCATCAACTCATATTATTTAAAGGACTTGTTTTCT
CTTTATTAGATTCTTTTTTTGTTAATCTTTGGT

>VfSat9_TA
ATAAAATAAGCGACATAATCATCATTTTAAATCTTTATTATTTTTTTCACTCAAATAGTTGTTATCTTTTCTAAAAAATGTTTTCTAAAAA
ATATACCACAAAAAAATCATAAAACTTTTTACTTGAAAATTCTAAAAACATATCATCAACTCATTTCTGCAATCACTAGTTGCATATTAAT
AGAACTTTTGACATGAAAGACAACAACTCATTTGATATGTGAATCATAACATTTTCGCATCAAAAGATTGACATATTTAAATTCTTATTTA
AGATCTTATTTGGGCAATGCATGCTGGTCAATTTGTGTTCGATGGCAAGTTGAATGTGAATCCCATGATGTTTCTTAATTGCATCAAATTT
TTCTAATGACGCTCTTACCTTGTGTGGTAAACCATGTTATTCAAACATTAAGGAATAAAATTGTCAAATTAGATTTAGTTAGGATCATGAT
TAAATGATTGATAAAATGACAAATTAATTTTCCGAGAGACTTTTAATAAGTGTATTAACTATTATTAAATAATAATATTTAGAAGATTTAT
ATTCACATTGATTCTTTCAATTTTTACATGATTATTCAAATTTTACTTAGATAAGGAAACTAAGGCATGTCGAATATACATTTGAAAATAG
AGAATTTAAAATAACTTGTAAAATCTATCGAATAAAAGTAATTAGGCCTTTTAGAATCATTTATTGACAATTTTTTTATAAAACTATTAAA
ATAATAACATTTGATTAATTAAATCATAATATCAACGCAAACTAATAAAACAGTTAAACAATGTTGTTACACGTAGCACTTTTCAACTCAT
AATATTTTATTTTAGATTAATTATTTTTAAGATTATATCCTATATATTTTAAAGTCGCGTAAATGATTTATTTAAAAAGTATTGAGCTAAA
TTATCGTGTAGGATTATTAATAATAATTTGATTTTTATATTAAAACAAATAAA

>VfSat10_TA
ATATAAATATTACTTTTGAAAAGAAATACATTTCACATTGTAATTATAATTTCAAAATAAGAGAGTTCAAAATTCATTTAAAAATGCACAG
ATTTCATAGTTTTAAAAACCAAGATTAAATTTCATTCAAGTGATAAAACATATTTAATTCTTATGAACGCACAAATTTCTCTTAAATTTCT
CTTATATCATATAGGCAAAAAAATATGATAACATTGCTCTACTTATAAATAAAAGACTTATAAATAAACTTCAAATGACTACATTAAACAA
CAACTTATTCTTCACATGCCATTAAATCAAAAGAGATAATATAGAAAAATTAACCTAGAATATTCATTACTCTAATAAACTTCAAAACTCG
CCACCTCACTTGATATAAAGTCCAATGATATGTTTAGAGTAATTCATAAAACAATAAAGATAGTTTACATAAAAACAAAAATAAATTATAT
GTAAATTGAGATGTGAACTCACATATGATCACACAGAACAAGTGAACCACCATCAAAGCAAATAAAATAAATAAGTTCTTATATTTTATCT
ATTTTATTGAAAAATATTTTCTTTTGGCAACGTTGTTGTCGCCGCCACTTCAGTATCAGAAGCAATGACTCTGATCATCTGAGAATTTTCA
AAATACTACCAATACCAATCGTTAGTATTATTGGTTGCCAAATCGGTTGAATATGATGTTCTTTCGATTGAAATTACTAAATTTTTATTAT
TTTTATTTAAATTTTATTTATGTTAATAAATCCAGTTAATGAGAATGCAATGTTATTTGTTTCCTATCAAGTGTTTCTATGTGGCTGTGTT
GCAAGTACAATTATCAAGTCTAATTTTTACCGTCTCTACAAAGATTGATTGTGTTTTAAAAACTGTTCAATTGTTTCTATAACTTTAAGAA
TGATTTATCAGTTTTTTGTGACTACAAAAAGTAAAATAACAGAAAGTAAAATGAACTTTGGTGTTTAACCGTTTAGAAAACTTGATGGTTT
AAATATCATTGACATATTATCATATAGTATCCTTGATCAGTAATATGCAATTCTAATCAATTATTAATATTACCGCTCGAAACTCTCCTCA
CAACTCATATATGAGTCAACGACGTGAGATTATTCATATTACCTAATATATGATATCTCAACCTATTAACCATCACGATATCTTTAAGTTT
CAACAGTTCATACGAATATTGAACCTAAATCTATGTCTAGACTTTAGCATATCCAATAGATGAAAAAGTTGGACCTAGTAATTACAAGTAC
CTTTCGTATATCAAGTCATACTATGAAAACATGTTTTAGGTAGATAATCCAAATCAAGCAATAAGAACAAATAAGTCATCAATGTTAAAGC
TTAAGAAGAATTACATAGAACGCCATGACCGGGATCAAAACATGAGTATTAGGACAACCTCAATTTTTAAACTAATTTGTAATATAAAATA
TAAACTTATTTTAGGTATAAAAGTTGAGAAAATGATTTTACAATAGAAAAAAAAATGCTATAATGGAAAATCTATGGAAGATGTCCTGATAC
TTATAATAACTCATACGTATGATATTCTTCTTAATCATATTTTACATTTGTTAAAGGTTAACTCAGAAACTCATCCTTCATAATTTTAAAA
TAAAATACTACAGTTGCTTTGAAATTCTTATATTTATATTTTATTTTTTGTCAAATAATTTAGTTGGCTTTCAGCCTTATTTTATTTTTAT
TTACTATTAAATTATTCTTCATAATTATATTTA

>VfSat11_TA
CCAGTTTAAATATCGATGTATGATTTAGATTAAATTGCATTTGTTTTTTGTTTTCTTCAATTTTTGTAAGTACAAATTTTTTTAAGTTAAG
TACGTAGGGAAATAGAAGTTAATATACATGTAAGTACAATTTTTGTAATAAGATGAAAGGAGAATATACAGTGATAAGTGTGATGTGATGT
TCATTGATGTTGTTTGTTATTGTTTTGGTTTTGATGGGTTTTTTTTCTTCATTTTTTTCTTATGTTGTTTAGCGACATTGGAGAGAGCGGT
AGAGTCTGAAGCAAAATGAATGAGTATAACGAGAACAAACCAATTGAAATCTTAGTTTGATTTGATTTTATATATTTATGTTACATTGGAG
AGAGTCTCTGTCCTACCTTAATTTTTAATGGTTCTTTTAGGATTGCGTGATTCCTTATACAATTTACATGTCTTTCTCAAATATTAAAATA
TGCATGTTTTTGTTTATCTAATTTAGAAAAACAAATAAGAGTATAACCTGAATAAATTTGTTTTATTATTTCTATATATGTTACAAATCAC
AAAGAGAGCATAATCACAGTTTAATGATTAATAAGTTATATATTTAGTTAGTTTCACAATATTTTTTTATAAAAATGTTAACATAAATAGA
TTATACTAAAAGGGTACTTTAAATAAATAAAATAAAAAATAAAAAATCTCAACAAAATCCAAATAAGAAAAATGAATTATAATGCATGCTA
TTTATACACTCATATGTATCAATCTGTTGCAAAGAAAAATGTGAAGCAAAGTAGCAGAAGGTATTTTAATACTTCTTATACACTCATATAT
TAGTTCAATTCTATTCATATTGAGGTTTAAGTTGTCATATTTTATTTTAAGAGTTCATCTGAAACTGACATATTTTTTTAAATATTATTT
TACAGTTTCTTTAAACCAATAAAGAGAACAATCGAATAAAAAATTACTATTTCATGATTTGGCAACCAAACTCACTCTAAAAGAATGCCAC
ATTGGTCAACTTGCCTATAACTATCATATTTTACATTTG
AAAAATAATGTATTTTGATTTTATTAAAATCTCTTTATAAGTGAGAGTAAAATGTAGCTTATATACTGCAATGTAAAATCAATTTTTCACT
CAATATAATGTGCAACTTGATTTGAAATGATACACGAGATGGAGAGACTTTTAATGTAGGGTAATCTCAATTTTCTAACTTTTAATTTCCA
TTGTTGGTATTTTTAAATCTTAAAGACATTTAAATGATAACTAGAATTATAATGGTCCAATTATCATTTAATATTATAATTGATGAGGATA
CAACTATTTATTACTCATTTTGATTCACAATGAATACATCTAAGTGAAAAATGAATAAATAAAAATGTAATGTTAAGTTTTTAAATATATA
TTTTATAAGTATGCAATTAATATAATAAAATAAATAGCATGCATTAAAATTATGTATAGTGTCTCTTCTTATTATTGTATATCTATCGTAT
AAGAAAACTCACTCCTTATTAATCACACCGTCTGCTTTGGATTCAAATTTGTCTCCTAACAACTTTGGTGAAAGACTAAACATTTATCGGT
AAATGCATCAGATTTGTAGAGGCTTCTAATTTT

>VfSat12_TA
AACTCGGAGACATTTTGATGAACAAGTGTCTTCACACAAACAAAAGTTAAGGGCTTTAAAAATTAATATTAACAATATAGTCTAGAGTTCA
TCATATGATATTCTTGATATTTTATTTCCTTCGTTGTAGTAATGGATTGTAAGAGACAACTTACATATAAACATCTTGAACACAATGTTTT
TGTTCATTAGTACTATTATTTTTTAATTAATTAATTTTTTTTTCTCAAAGCTAGTTTACATCCAAGGACCTATTGGCATGGGCACACATCCC
TTCAACATCCCAACTTAAATTTTCATTAAACCTTAGAGGTTGTGTTATAAAAAATTCTAAAAGATAAATAAAATAAATAGATATCTTCACA
AAATATTCTTATTATTTTCCATAATGATAAATATAAAAGAATTAGAACAATAAATATTTACCTTAAACTTATAATTTCATATCATTAATAG
ATTAAGCATTGGTAACATAAAAAAATAAGTTTCATTTAGTAAAGAGTTTTCATCCGTATAACAATGAGTAATGAACCATTTAATTTATATG
CATCTTTATGTATGGTGATGATCATGCTAGTTCTAGAATAACAAACAACCAACATAAAAAATCTATTTCACTTACACACGTAAAATTCATA
ATGGGTGAATAGTCGAATATGAATAAATATGAAACATCATATATATGACTACCGTCACTAGTAAGTATTCACATGACATTTTATAATGACA

TAAATAAATATTTCTCGTATATCAACATATAAAATTTTTTGTGCATTGAATTTATAGACACACATTTGAACTGAAACACATATCCTTAATT
AAATGTGAGATAACAACTACGGACATACGACTACATATAACTAGTTATTGCACATTGAAACCAAAAGTATTGTTCCATTTATTGCAAATGA
CATCATAAGTTACACAAGTAATTTTTTTCAGTATTCATACATGTCACTTATATTCATTATGATGAACGATATTCCAAGCAAAACGTAGTTA
GAT

>VfSat13_TA
TGCTTGTTTAGGAATACTTATTGTGATGTCTTGATTCACTTTCCTAT

>VfSat14_TA
TTTAGAAATCTTCTATATCAAATATGAGTGTACGGGAAATGACTTATTCATTTCCATTATTAATATACATTCTGAAATATTATTATAAAAA
TAATTATAAAAACTAGCTAAGAAAAACAAAAAACAGAGTAAAAAATTGAAAACAATTTGTAAACAAGTCACCAAATTTAAGAAATTTAAAA
CATAATATATTTAGATAAAGAAACTAATCAAACAAAGTCAAAATTATAATACTTAATTAAAAATACAAATTTATTGTTGTTTGTTGTTTTC
CCTTATTATATTTTTTAATTTTTTCTTTCTACCTTTTTTTATTCTATGGACGAACTTTTTTTTGTTCACTTATTTTTTTTATAATACTTAA
TTTTGATTTTTACGTTATATTGGATTTTAATTGAGATTTTGATATGTGCAATTGCCTGCCATTTGCGATGGTTTTGATTTTTATGCTATTT
TAGCTGATGATTTTGATATGCAACTTACGTTTATTTTTTTTAATTGAATGTGCTTTGAGTATTTTTACATATCCTTGAATTGTGATATTTG
ATAGGATGAGTATCACATTAATTTTTTTAATTTAAACATCACAGGATTTTTGAATGGTACCTCTCTTTCTAAAGATTTGACAATGAAGAAG
AGAAAAACAAAATACTAAATAGTAATGCAAAAGATATGCCGGAAGATATTAGGGAGAGAAAGGAGAATTGCCCTATGAAGCAGGTTGGAAG
TGCATTTGATTGGAATATATTCAATAAAATAGGACGATTGAGAAATTGAACAGAGAGTAAAATTTAATGCAATTCTAATGATTATATCTGC
GAATGCCTGAATTTCCATTTTTAGAAACATAGTGATTATTAATCTTTAGAAACATAGTGGTTATCAACC

>VfSat15_TA
ATTTTTAAAATAAATTTTTAAAATTTAAATAACACATCATCTTGAATTCTATTCACCTAAGATGACACAATTACTAAGAGTCATTATCTCT
GATTTTGCAAAATTCATAGTAAATTCTAGCTTCTTTAAAAGTTTGATAAGTGTGTGTCTGTGAATATAATATGTAATGTGATATAACATCA
CTTTAAAATATATACAAACAATATGAAGATCAATAGAGAAGACAAAACATATTAGTGTGTGTCTTTAAAATACTCAAATCTTTAAAAAAAA
TCATTCATATTCTAATGAATCATGCAAACAAAATAATAATAATACACAATATAGAGATATGTTTGTTAGAAAAATTAGAATTGTTATTCAA
ACAAGTTTTATCTTAATATTTACATATACGAGAAGCAAAAATAAGAAGAAATCTAAGATTATTTGATGCAAAAAGAAATACATACTATTGT
TATATTTATCATCTTCCTTAAGTTTCTCTTTGTGCAAAGTATAACAATGACATGATCAAATTAAAGATATGACATAAATCACCTTTATGCT
GAAAGAGTGTCCTTTTTTTATGGTGTTTTTCATCATAATATATAGTACCAATTTCTATAATAAGTTTAGTTACTATAATTTTACTACTTGT
AAGAGTTATAAGTAGTTAGTTTCTCTAGAACATAACAAGCAGTCACTTTTATGACATCTAGTAAGTGTTGTATAAATTAAAATTGATAAAT
AAATATATTTGCTCAAAAATTGGGTAACATTAGTTAAAGGTTTTGGCTTTCTTTTTTAAACATTTCAATGTAAGAAATTAAAATTAAAAAC
TCGTGTAAATGCACGATTATAGAGTTTTGAGAAGTCGTCCATCCTGACATTGCTCAAGACCACTACTAATACAACTTTATATACAATATAA
TTCTTCAAAAAAAATATTTTTTTAAAAAAAGG

>VfSat16_TA
AAGAAGGAAAGGAAAAATTTCGAATAAAACCCACAAACAAAGGATAAGATGGTCTTCGAGACCAAAGAGAGGGTACATGAGTCGGTTATGC
AAGGGGAAGGTATTAGCACCCCTCACATTCATCGTACTCGATGGGAACCATTTGGTTCGTGTGTGTGTGTTCGAGTGGTAGTGTGATAGTT
TGCAATCTTCTACTTATTAATCTTGAAAGGAGAAAGAAGTAGGCTTTTTGTTTTTTAGTTTGTTGAGTTCGACAAGATTCGCATCTTGTGT
CTACGTACTCCCTCGTGCAATGGGAAAGTCAGAACTCCGTAGTTCTTCTAAAAAAGACCAACGGTGTATTGCTTGATTTTAGAAGAATGAT
GAGTTAGACATTTCAAACGTTTGAACTTCGACTTGTTTTGCTCGTTCGCGGAAACTAAGTCTTTGTGTTTGTTTTCCTATTAAAATGGCTA
AAACACATTCCTTTTATGAAAAGGTTTTTGATGTCGCGCAAGGGCGAAAAAACAAGTTTGATGAGTTGAAGTTGTTTTTATGTGGGTGATG
AGTACCGAAAAATCGGACTAACATCCTACGACTCAAATACTAGAAATTAGAGGATAAATGAAGCTCAAAGAGTAGTCTCTCAACCCCAAAA
GTTATTTTCTTATGAAAAAGATGAAGTGAAACAAGGTTCACGCTTATTAGGTTTTTTGCACGAGTCTTAGACAATTGGTCTAACAACCAAC
GATCTAAATACTCAAAATTTATTATGAAAATGTTTTTGAAAATATAAGTCGACGTTGGATCGAGGATTTGAAACTTTATTATGAAAGTGCT
TTAGATAGAGAATGGGAGAAAGTCGATTGCGGTTGCAAAAGCAAACTCGACTTATCAAATTAGTTATGAGCTTCGTATGTGGACCAAGAGT
GCACGAGCTAGATTGATTCAATTAAGTGTTCAATAGTGAATAAGCGATTAAAAGCAAAATAAAACTATTAAGCTATTACACGTCCAAAATG
CAGGGATACACTTGTTTAATGATGATTGACAAGTAAATCACACAAGCCCATACAAGGTGGCCCACACAAATGAAAAGGATAAAGCAAAAGA
GATGAATTATAATCTCTAAGTGCACTAGCAAAGTGGGCTAGTCTTCTAGACGACTAATTAGAATAATTAACCGACTAGGTCTCACTGTAGG
AAAGCCCAGGATTAAGCTATGAATGTGTGCAAGTGTGCATGTGTTTCGGTGTGGAGGGGTCCAAGAGGAAGCCATGCGAGGTCGTTTGCGA
TGCTTA
GAAATAAATTGACTTATGGTGGAAAATTGGCTCGCATTCGAGCATTAGTTCTTTGAAAGGTTCGATGCATGATGATGATGAATGATTAAGG
TAAAGCAATAAAGTACAATTAAATGATTATTACATCACACGGGGATTGGGTACAACCTTTTGAATGGGGATGGAACCAACCAAATCAACCA
CACACGAAAGCCTAATTAAAATCAAACAATACAGACCAATTGAACCAAATAATTAATTAATTAAACTAAATAATTTTATTTAATATAATTT
ATTATTAAATTAATTAATATTATTAAATAAAATTGCTTAAGATAATTAATTATCTAAACACACGTTTTTTGTATTTTTATGATATAAAATA
AAAAATAAAAAGGAAAGGGACATGGAAAATATAAAACAATGTAGTCCCCGCCGAGATTTATTTATTTC

>VfSat17_TA
GGTAATTGTATCACACGCTTTTATTTTATTAATAATAAAAAAGTAAGTTTTTTCTACTTAACTTAAATTCTTTTTTAAGAAGGAAAGCATA
CATTTGGTATGTTTGGCAGATGATGCAGGGATGAATATCTTCACGTTGAACAATTAATAATGGATTGATTTGTTTTTCTTCTAATAACTTC
TATGTTGTAATTTTTTTGAAATGTTTCTTATATAAAGAATGAGGATAATATTAAGCTTTTTTTTATTAATCATTGACATGTCACACATTGTAG
ATAGAGTAACACACAATTAAATGTGAAATGCATATTTCATTCGAAACCAAATATCTTTTCTATAATATAGGTATGAGAATGGTGTATCTTT
TATCAAATGTATCCATTTAAAAACACATTAATGATTATATCTCATTTGTTGTAAACCTTATCTTTTTTTGTTCAAGAATCATACTAATTATC
TAAGAAAAACACTAAGAAGTGGTGGTATCAACAAGTTTCTTAGTGTCCATGTCAATGAAAAAGCACATAAAATGTTATTCTGTATTGATTT
CAACCTTCACTCGCTTATGTTTAGTAATAGTCTCATTTTTATACTATTTAATTATTTTTTTAAGTATTCTGGCATCGAATATGTGAAAATC
TTACAATTTGTACTTTAAAACAATGTGACTAAAAATTAATATTTATTAAATGAACATGCTTTTAATATTGATAGAGTATGCATCCTCTCAT
CTTTGCCTAAAAGAAATATCAAGAATGAAAGGTTACTATATGAATAAATTAAA

>VfSat18_TA
AAGAATAAAATTTAATAAGAATATAAAAAAGAGTTTTTATTTTTAAGACACAATAGGTCACACAATAAGATTTTTTTTTTAAAAAGTCATG
CATATAGAATATATTTTAACTTTTATGTCAAATTTATTGAAGTTATTGATTTTTAGGTATGATAAAAACAAATTTTATATATGATAACAAT
TTTTAGCTCTTATTTATATGATAAAAACTATATATGCTTATAATAATTTTTTGGAATAACTAAAAATTTATTTTTCTTGACCATTTAAGTT
TACTAAAAAAAATAACACAATTTTCTTTACAATTTAAGGACAAAAAACACTAATATTTCTAAAGACACTAAATGAATTTCGACTCCTGCCT

ATCAATCATTTCTTCCTTTTTTTTAACTGCATTTTGTTTCTTACCTTTCATTTTCATTTACTCATAAATTTCTCTGCAATTCAAACAAATA
TCCAAATTCTTTACAAAGTGATTTCTCAAAATTTTTAATTCTCATTCATTAAATCTTATCTTAAATTGTGAATCTAAGATTCATCGGAAGT
ATTCCTTTTGAAATCACATTTATGGTTTTTAGACATAATTTTGTTATTGAATTCACTGCAATTGCGCTAAGCTACTTTAAATTAGAACAAT
GTGGTTTTGGTTTTGATATGATTAAGGTTGTCAGTTAGATGATGCATTTTAGTTATATCTTAAATGTAATGATATTGAATCTTTGAAGATA
TTGTAAATAACTATTATTACCTTACGGGAAAAAGTGACATAAAATTTTATAAATAAATGATAACCACTCAAAATAGGATTGATATTAATTA
TGTGTGTGATTAGTTGAAAAAAAGTGGTTTATTAATTCAACAATATTTCTTTTCTTCTTTACTATATTACTTGTTTTTAAAATATGTAGTT
TATTAGAAAGTTTATATAAATTAATTTAGTGAGTATGCACATAAATTATTAATATGTTTAATTTTTAAATATTATAAATGTTCAAACTATA
TGTTATGTTTGTTATGAAACTTGGGATCCAAAGAAAAATAGAAAAAGTTAAATAAAATATTGTATTTTTGTTGATAAATATAATTTTATTT
ACTATATTTTTCCTTATTACATAACTAAGTAGTAAAAATGATATATATTGAACAAAACTTTAAAAAAATTATTATTTTTC

>VfSat19_TA
TTTTAATATAGATTTTATTACCAAAATGACATGTGACCATAATTATTGCCAAAAAATAACATGTGGCTACGGTTGTCATCATGACTTCTTT
TAAATTTATGATAAATAGATGACAATTGTAAAATCTTATAATGTAGTAATATTTTTTACATATATTTTAAAGTTGTAGAAACGCACCATAT
TTGTTTATGCAAAAAAAAAACATTTCTTGAATATTACCTAACAGATCTTATTTATTGTTACATATTTAATATTGTCAATGTATCTTATATA
TGGTTACATACTTAATATTGTAAATGTGTGAAAGTGAGATGTGGGCCGATGAGGGAGAATTCTCACAACACATTGTTATTATAGGGATTAG
GAATATATAAGTTATCCTATTGGGCAGTGTTATTGAAAAATTGAACATCGTTAGATTATAAATAAATAAATATTAATATGTTATACTTTTA
TGATGACTTTCTATAAGATTTATATTATAATTATTGTTGTCAAATTTTGTCATTTCTAAATATTTCCTAATTATTTAATTGAGTGATTTTA
AATATTATTTAATAGAAATTTTTGTAAATAACATATAATTATGTTTTTAAATTTTTAAGAGTTGTAATTTGCATATTTAAGTTTAAATAAA
AATCATTTAAATTCTTTTAAAAGAGTTCAAAAACCTATTTATAAATCATTTAGGCTTCTATATAATAGACGACTTTATATTTGTTTATAAT
AAATTAGTCGATTAAATTGTATATTGAAAGAAAAGAAAAATATAAAGTTGTTGATTTGTTTTATTAAAAAAAGCTATAAAATACAATTATA
AACTTTTTTGGGTATAAAATAACAAATAACCATTCATTTACTTGTGCAGAATATTTACAAGTTGATTGAATTTGAAGTGTATGATAATTGT
AATGGTTGAAGCCACTTATAAATCTCAAATGACATAAAAAAATAAGTTTAATTAAATTTAATATTTTAATTAAGATTCTTGGAATTAAGCT
GAGGAAAAAAACTATAAGTTACTTGATTTAGTTTATTAAAATAAACTATTAATCAATAAAAATAAATTATGAACTCTTAAGAAAATGATAA
TTATTAAATATATTAATTTTTCCAATTATAACGCAAAATCTATTAACTATAAATATAACTTTGTCAAAAGAGAAATAAACACAACTGTCTT
TTGCCAACAAAAACCTATAAGTTTGATATATAGAATAGCTGATAAACCATATTATTAATAACAAATAAATTAGTTTATTGAATTTGTTGTA
TTTAATAAAATTAATGGTTAAGTTAACTTGTATTTTTAAAATAGTTTTATTACTATTATTATTATTATTTA

>VfSat20_TA
AAACTATATCTTTCTTTAATTGTGTTGGCTAAAATACACTAATCAATTTGTCATTCATTTTTAATTTGTAATTTAATATTTTATTATCTGA
AATCTTTTAATTAACTAATTATTAACTAATTTAATATTTATCAAAAATATTTTAATCAATTAATTTTTAACTAACTTAACTCATCATCCAT
TGGAAGATTAAAAGACATATATCACATTTTTTATAAAAAGATTCAAATATGTAAAAAAAAGAATATGAAATAAATTAAGGAGAAAACATGC
CTTCGATATCTTGCATCTGATTTAAGAATGATTGAACCATATTCATATAGATGTTTCTTTTATTCTTCTATTGCATCCGTTTTTGTTCACA
TTTTTCTTCAAGTCCTCTTTCAACCAATAGATTTCATACTGTTAATTCCTCTTCATCAAGTACTAAATCAACTCTTGAAGATGGTCGTTTA
TAGATAACTAACTCCCTTTATTTTTTGGCATCTAACTTATTTATCTTTTGTTGTTTTTTCTTCTTTATATAAGATAGTTTATTTATTATAA
GTCTCATTTTTCTGCCCAGTCTAGAACTTCCTTCTACATGTAAAATTTCAAAATATTTTCTCTTTTTCAACAAAAGACTAGATCTTTTCAA
CTTTGCAAAAGGGGTTAGCACTTTTATAAAACACATTATTTGTATTATAATTCTTTTCTTCTGATCACAACTAGAGACAAATATGACAATG
GAGTAATCTTCTGATAACTTTGCAGAGAATTATATTCAATGGCATGTTACCATGATCAATGGTTCAAAAATTGATATTTTCTAAAAATGGT
AGAGATATGAAAAGTCCATTATCATCAAATAATGGTCGCAGACACTTATATGCTCTTTTGACATATGGTTTTATTTGCTTCGGAGTTGAAG
GTTTTGGAGTGAAA

>VfSat21_TA
CTTTTAGATTTTTAAACGTCAATGATAGCATTAAAAAAACAAGAATTTGAGAAAAAGAAAATAAACTTTTGTAATTTGAAAGTAGACGATG
ATTCATGGTGGTGGTAGGAAGTCCATGATATGAGAAAAAAACTAAAAAACATTTATGTGTGAGTGGTACATTATATCATTTAGTACAATCC
TTTAATCTAATGGATCTTTTCCATGGTTTATCATGAACCATTTAGGTGACGACTAAAATTTTAATGTCTTTTATCATTATTTTGAAATAAA
TATTTAAAATTATAAATTTTTGACTATCCCTTATAAAGTTGAAGATAATTCATGTCAAATGAGTATCAATAAATTTATTATTATTTTACAA
ATTATTTATGCTAACTTTTTGGATGAGGGTTCAATTGAATCTCTCATGTCATCAGTGAACAATTCAACTTTACCATAAAATTAAACAAGTC
TTCATTATATATTTATATATACTGTATTAGATTAGATCTTTCCATGCTACCCCATTTTGTATTTCTGTAGTGACAAATATGATCACACTAT
TGTAATCTCCTTGCAAATTGATTATTATTGTTTTAGAATATCAATCCCATCAGAACAAAGCTAAGGCTCTGTTAGTTCCACCACCACATAT
TATTATTTTTATTTTACTTTAGACAACAAGTACACGACCTATGATTTTTTTTTGGAATCTAAATCTTTCCTTTTATGTTTTTTCAACTATA
CATTCATGCACGCTTCTATAAAATACCAAAAAACATATACACTAGTATATTTATTTATTTTTTCTGTTATCTAACTTGCCTCCTCTATTTT
CATTTGATGTAGTTTTTCACCCAAAGCATTAAAATATAAAAAGAAAATAGATGCCATAATATCAAATTCTAAACGTATTCATGATAACAAA
AATGTCAATTTTTTAAAAACAATATTCAAAAATCATTCAAGATGGATAATGAAATATAAGAGTGACACATTATGAAGTACTTAACATCATT
CAACCACTCACAAAGCTTGATATAATTTCCCAATCCAAA
ATTTTTATCAACAACTT

>VfSat22_TA
GATTATTAAGTACATCCACCTATCTCCTTTTAATGCTTTCTGAGTGGGGGAGATTACTAAGTAGACCCACATATCTGTCATTTGATGTTTT
CTCATATTACAAGTTCAATACGAATGATGTTAAAATATTATATGTTTGCCATGTTAACACTGCAAACATACTTCATACTGTATATTTCCAG
TTCATTTATACTAAAAAAAAATCAATTAAATAGTCAAACTATTTTTTCTCTGTTTATATATTCAGTAGACGGAATTTATTATTCAATTTAA
TTTAAGATTTCGCTTTACTTCCTTATCTAAAAATCATTAAATTGTTCTGTTAGCATTATTGGTCCCTCCGTTAACTTTTCTGAAAATTATT
AACTTTTGCTAACATGGCATGACAACTAGGATACTCAGTCGGTAAATATATTAACAAAATTGATATTTTATATAAACTTGAGAGTATTGTT
TTCAGCGGTTAAAAACCCCCACAAATTGTTATTAATGTGTAAAAAAAAAGAGACAATTATTCTCCTTTCATTAATAATATAATTATTCTCC
TTTCAAATTTCAACGCTCCTTCAAGTATAACTGCATGTAAATATATTCATACATTAATACAATTCTTCTCCTTTCAAATTAAAAAACACTT
GGAGCTTTAAATACACCAAATACATAATTCAAAACAACACATGACAATAATGAAATTATTCAATTGGTAAATTAATAAAAATTGTCATCAA
GCAGAACAAGCTTGAGCAGGAGCCTCAACACTTTCATAATTAATGATTTCTTGTTACAAACACTTCATTATATAACCAGATTGACATGTTT
CTTTTCCTAAAACAAACACAAAATTACATTTAAGTAAACAAATAGCTACTATTCGCTTGGATTAGTGACAAATAGTGTTTCATATTTACCG
AGCATCCAACATTGACAACACTGCATTAAAAATCAAATACGACAACGTATTTAAGCAATTAAACCTCTGTATTACAAATTTGGTAATATGA
AATTAACTCATAATATATCTAATTGCTGAAACAACTTAATGCAAATAACAGGGTTGCAACCACAACTTGAACATCTGGTTGAAGGAGAACT
TTTTTAAACACATCAAACTTAAATTCCGATTTCTTTTCATTCTCATCTTGTGTTGTTTGCAACCTATAATCTTCTGCAAACAACCCAAAAC
AACATAAAACTCATAATAAATCAATAAAAGAATTTTTTTCTTCGATTTGATTCAGGAACCACAAACCCCAAAATTTGCATCAATTTAAAAT
TATCGGAAAAAAACCCTTAACCTTAACCTTTTTTTGGAAAATTAAATAAAACTTAAAATTGAAAGTTAAAAGAAGATCCAAAATAACTAAT

```
CCAAACAGCGTTACCAACCACATCAAGGTAAGTAAAAGCAGGAGGAAGATGTAAAAAAGAAAACAAATGAAGATGAAGGATGGGAAGCAGG
TGAAGAGTTTATGGATGAGAATGAAGAGAAAGAGGGTGACGGCTGCTAGGGTTCTTGGAAGAAAAGAGAAAGAAGGTTTTCATAAATATCA
AAAAAACCACGTGATACGTTAAAAATCTATCACTGCCACATATGCAATAATTAACGTTGATTCTTGAAATTTAACAGAAATGACTAAAATA
ACTAACGGAAAAACATAAAGAAATAAAATAATAAATTAAATTAAGAAATTAAAAGATGAATTAAGTCGAATAAGAACAATCAATTTTATAG
AATAAGAATACGTGTTGATTTTTACATTAAAAAAACTTGGGGAATTTTTCTATTTCAAAAAAAGTATTATTAATCTTCATGCTATATTGGC
TAGAATGGTGGAGA

>VfSat23_TA
TTGAGAGATTTTCATGAAGGTTTGCAATTATTTAGAATGATAATGGTTCATAAAAAGACATAATTTTGAATTATGTACATGTCTTATTAAG
TCGGTTAAACGGCCACTTGATTTAACAAGTTTGCAACATTTTTTAAATTTCAAATAATTTTTCAAATTTCAAATAGCATCAGGTTTTATTA
AATCGGTTAATCAGGCCTGGCCTAAAAAGTGAAAACGACCTAACTTAAATATTTCTGGCAGCTTTTTAAAAATCAACTATTTTTATGCCTT
TATTTCAAAGATTTTTATGCATGACAATTTGAGAACTTTATATATGAAAATGTATGAACTACTTTGAGCATCTAAAGTTTATACATAATGA
ATTATAATTGTTTACCTTAGCATCAAGATCAATTTCCTTTACTTCCAAAAATATCCATAAGCTTATGCATTCTTGATTCCAAGCTGTTTCT
TCTTTGACATTTCTTTTTTGATAAAATTTTGTCTTCCTCAAAACTAAATTAAAATAGATGATGAACATCTTCTTCACAATGATTTGATGTC
AAATTTGATATTAAAGTTGATGCTTCTTCAAATCATCTTTCATTCAATGATATTTTTTGGAACTACTATATCATATGGTGTTGTATTTTGA
AGGATCAATCATGATTTGACAATATTAGCACATGATCTTCAAGTACCTACAAAAAATTAGTAATACTTAGGTACCCAATTTTCATTGGGTC
CTCTTGGGTTAGATACCTTCCTCACCCATACATACTCACCATAAGGAATACCATAATTTCTTACGTTGCAGACATTATAAGTATGGTCTTT
TGCATTACAATAAAAACAAATAGGTCTAAAAACATGATTCTTTTTATTGACTTAAAAGTTATTTCTATCATAAGGCTTTTTATGATGATTT
ACAACACGTTTTTTCTTTGAATCCTAATTGTTGAATTTCTTAGTAGCCTTTAGGAAGATAGTTGGACTTGTACTAGGTTTGTCAAAATTAA
AAAATTCAAGTCCAAATTTATTATTTGAAAATATTTGACTACATGATACATTATCCAAGCTAATTTCCCCTTTTTCATATTTTTCAACGAC
TTGGTTTAATTCCATAATTTTTTATTCAAAATAAGAACAATTATTGCATGCTGAATTTTTTATTAAGTCTAACTCCACTTTTGTGATATTA
ACCTCACTTTCTAGATTTAAAATAGTTTTATTTCGATTTAAAAATTTTCTAAAAAGTTTAAAAAAATCATCATGTAATTTATTAAAAGCAC
TTTGTAATTCATCATATGAAGGACTATCATCACTTGAGTTGATGTAAAAGT
```
